# Oral Administration of East Asian Herbal Medicine for Peripheral Neuropathy: A Systematic Review and Meta-Analysis with Association Rule Analysis to Identify Core Herb Combinations

**DOI:** 10.3390/ph14111202

**Published:** 2021-11-22

**Authors:** Hee-Geun Jo, Donghun Lee

**Affiliations:** 1 Chung-Yeon Central Institute, 64, Sangmujungang-ro, Seo-gu, Gwangju 61949, Korea; jho3366@hanmail.net; 2 Department of Bioinformatics and Statistics, Graduate School of Korea National Open University, 86 Daehak-ro, Jongro-gu, Seoul 03087, Korea; 3 Department of Herbal Pharmacology, College of Korean Medicine, Gachon University, 1342 Seongnamdae-ro, Sujeong-gu, Seongnam 13120, Korea

**Keywords:** association rule analysis, complementary and alternative medicine, East Asian herbal medicine, meta-analysis, peripheral neuropathy, systematic review

## Abstract

This review aimed to comprehensively assess the efficacy and safety of oral East Asian herbal medicine (EAHM) for overall peripheral neuropathy (PN). In addition, an Apriori algorithm-based association rule analysis was performed to identify the core herb combination, thereby further generating useful hypotheses for subsequent drug discovery. A total of 10 databases were searched electronically from inception to July 2021. Randomized clinical trials (RCTs) comparing EAHM with conventional analgesic medication or usual care for managing PN were included. The RCT quality was appraised using RoB 2.0, and the random effects model was used to calculate the effect sizes of the included RCTs. The overall quality of evidence was evaluated according to the Grading of Recommendations Assessment, Development, and Evaluation. By analyzing the constituent herb data, the potential association rules of core herb combinations were explored. A total of 67 RCTs involving 5753 patients were included in this systematic review. In a meta-analysis, EAHM monotherapy and combined EAHM and western medicine therapy demonstrated substantially improved sensory nerve conduction velocity, motor nerve conduction velocity, and response rate. Moreover, EAHM significantly improved the incidence rate, pain intensity, Toronto clinical scoring system, and Michigan diabetic neuropathy score. The evidence grade was moderate to low due to the substantial heterogeneity among the studies. Nine association rules were identified by performing the association rule analysis on the extraction data of 156 EAHM herbs. Therefore, the constituents of the herb combinations with consistent association rules were Astragali Radix, Cinnamomi Ramulus, and Spatholobi Calulis. This meta-analysis supports the hypothesis that EAHM monotherapy and combined therapy may be beneficial for PN patients, and follow-up research should be conducted to confirm the precise action target of the core herb.

## 1. Introduction

### 1.1. Description of the Condition

Peripheral neuropathy (PN) is one of the most common causes for a patient to visit the clinic [1]. The prevalence of diabetic peripheral neuropathy (DPN) or herpetic neuropathy, the commonly observed PNs, is at least 10–20% [2,3]. However, it is not easy to collect the available PN epidemiological data since the causes of pathology are very diverse. In addition, the symptoms can develop not only in a single affected area, but also in multiple nerves [4]. Symptoms that may occur due to this disease include chronic pain, decreased nerve conduction velocity (NCV), sensation loss, and abnormal sensations such as tingling, burning, and numbness [1]. However, PN pathophysiology is not clear. Moreover, its symptoms are not easily improved and often follow a chronic course or worsen continuously [4]. Therefore, the medical management of this disease is challenging due to the various characteristics of PN, which are difficult to manage and reduce the quality of life in patients.

### 1.2. Description of the Intervention

Several epidemiological studies have reported that the treatment results for PN patients are unsatisfactory [5,6]. This is primarily due to the fact that accurate PN diagnosis and management are difficult, and the prognosis is poor. Moreover, it is a reminder that the development of effective medications and therapeutic tools is urgently needed. The East Asian herbal medicine (EAHM) deserves further investigation as a potential pharmacotherapy for PN since it has long been providing benefits to patients with neurological and painful disorders in Asia [7,8]. Recently, several studies have examined the safety and effectiveness of using plant preparations for neuropathy to confirm the advantages of compliance with high-dose treatment, few side effects, and safety even during long-term administration [9]. Furthermore, the number of scientific studies verifying the efficacy and safety of East Asian medicine in PN has significantly increased over the past decade [10,11]. Previous systematic reviews have comprehensively dealt with the effectiveness and safety of acupuncture interventions in East Asian medicine for treating PN [12]. However, only few systematic reviews have focused on the association between EAHM and PN subcategories, such as DPN and chemotherapy-induced peripheral neuropathy (CIPN) [13,14].

### 1.3. How the Intervention Might Work

Several EAHMs with pharmacological activities against PN have been reported. A previous study has reported that various herbs, including EAHM, relieve neuropathy symptoms through serotonin 5-HT1A receptors, inhibit axonal degeneration, improve axonal transport, and suppress TNF-α and NO in CIPN [15]. In contrast, the Huang–Qi–Gui–Zhi–Wu–Wu decoction, an EAHM prescription widely used for a long time, can improve CIPN by controlling the inflammatory response and repairing nerve damage [16]. Radix Astragali, one of the most extensively prescribed herbs for chronic pain, including neuropathy, acts as a potential nerve growth factor to induce axon growth in peripheral nerves and promote nerve cell differentiation. Astragaloside IV, one of the main active ingredients of Radix Astragali, contributes to sciatic nerve regeneration and functional recovery in mice [17,18].

### 1.4. Why It Is Important to Conduct This Review

In the past decade, numerous randomized controlled trials (RCTs) have been conducted to assess the efficacy and safety of EAHM for PN. In addition, studies on drug discovery, which can regulate neuropathic pain based on EAHM, are actively conducted [19]. Several systematic reviews have already focused on this topic [13,14,20,21]. However, unlike acupuncture, a study comprehensively reviewing the efficacy of EAHM for PN has not yet been published. In addition, the EAHM prescriptions used for the individual RCTs included in previous reviews are heterogeneous, and a single dose and composition of herbs are not often utilized. Therefore, it was difficult to derive useful pharmacological information that can be used for follow-up studies or clinical practice in a previous review. Separately, although most of the herbal medicines have been orally administered in East Asia, whether studying different formulations, such as injection or topical formulations, in one review are appropriate, is controversial.

Therefore, the aim of this study was to comprehensively assess the efficacy and safety of oral EAHM in overall PN with multiple underlying causes. Additionally, an Apriori algorithm-based association rule analysis was performed on the various herb data to identify the core herb combination, thereby further generating useful hypotheses for subsequent drug discovery.

## 2. Methods

This study was conducted in accordance with the guidelines of the Cochrane Handbook for Systematic Reviews of Interventions [22], as well as the Preferred Reporting Items for Systematic Reviews and Meta-Analyses 2020 statement (Supplementary Material S1) [23]. The protocol of this systematic review has been registered in PROSPERO (registration number: CRD42021252277, available from: https://www.crd.york.ac.uk/prospero/display_record.php?ID=CRD42021252277, accessed on 9 October 2021).

### 2.1. Search Strategy

A comprehensive electronic search through four English databases (PubMed, Cochrane Library, Cumulative Index to Nursing & Allied Health Literature [CINAHL], EMBASE), four Korean databases (Korean Studies Information Service System [KISS], Research Information Service System [RISS], Oriental Medicine Advanced Searching Integrated System [OASIS], and Korea Citation Index [KCI]), one Chinese database (Chinese National Knowledge Infrastructure Database [CNKI]), and one Japanese database (CiNii) were performed from inception to July 2021 by two investigators. The following Boolean format was used for the search: (mononeuropathy [MeSH] OR nerve compression syndromes [MeSH] OR neuralgia [MeSH] OR polyneuropathies [MeSH]) AND (“neuropathy”[Title/abstract] OR “peripheral neuropathy”[Title/abstract] OR “neuropathic pain”[Title/abstract] OR “neuralgia”[Title/abstract]) AND (“Medicine, Chinese Traditional”[MeSH] OR “Medicine, Kampo”[MeSH] OR “Medicine, Korean Traditional”[MeSH] OR “Herbal Medicine”[MeSH]). In the Korean, Chinese, and Japanese databases, these search terms were appropriately modified to perform a search. The detailed search strategy has been explained in Supplementary Material S2.

### 2.2. Inclusion and Exclusion Criteria

#### 2.2.1. Types of Studies

Only RCTs evaluating the efficacy and safety of oral EAHM administration for PN were included. There were no restrictions on language or publication time. A few studies were excluded if they met the following criteria: (a) Not an RCT or quasi-RCT; (b) the control group was not used or was inappropriate; (c) unrelated to PN; (d) animal studies; (e) review; and (f) not published in peer-reviewed scientific journals, including postgraduate theses or dissertations.

#### 2.2.2. Types of Patients

All of the adults (age > 18 years) diagnosed with PN were included without restrictions on gender and nationality. The types of PN were classified into diabetic, chemotherapy-induced, postherpetic, and other causes, according to the underlying pathology.

#### 2.2.3. Types of Interventions

All of the EAHM forms, such as decoction, granules, capsules, and a combination of EAHM and another active treatment for PN management were included. The mode of delivery was restricted to the oral intake. Studies in which East Asian medical interventions, such as acupuncture, massage or non-drug therapy, were only combined in the treatment group were excluded. Studies in which the comparators included other EAHMs were excluded. Moreover, studies that exemplify the details of herbs constituting the revealed EAHM prescriptions were excluded.

#### 2.2.4. Types of Outcome Measurements

Primary Outcomes

NCV: Improvement in NCV measured in each body part.

Response rate: Rate of improvement or no improvement in symptoms, such as NCV, pain, numbness, tingling, and weakness.

Secondary Outcomes

Incidence rate: Occurrence rate of PN due to multiple underlying causes.

Pain intensity: Intensity of PN related to pain symptoms, as measured by instruments, such as the visual analog scale (VAS) or numerical rating scale (NRS).

Toronto clinical scoring system (TCSS) [24].

Michigan diabetic neuropathy score (MDNS) [25].

Adverse events (AEs).

### 2.3. Data Extraction

Two review investigators (H.-G.J. and D.L.) extracted the following information: (1) First author and year of publication; (2) type of underlying cause; (3) patient characteristics, including sample size, gender distribution, age range, and disease duration; (4) intervention group; (5) control group; (6) treatment duration; (7) main outcome measures and intergroup differences; (8) AEs; and (9) detailed EAHM composition.

### 2.4. Risk of Bias in Individual Studies

Two review investigators (H.-G.J. and D.L.) independently evaluated the RoB of the included studies according to the revised tool for risk of bias in randomized trials, RoB 2.0 [26]. Disagreements between the two reviewers were resolved through discussion. R version 4.1.0 (R core Team (2021). R Foundations for Statistical computing, Vienna, Austria) was used with the ‘robvis ’package to generate graphical presentations of biased risk assessments [27,28].

### 2.5. Statistical Analysis

#### 2.5.1. Meta-Analysis

For continuous outcomes, the mean difference (MD) was calculated with a 95% confidence interval (CI). A standardized MD (SMD) of 95% CI was used to express the intervention effect when the same outcome was measured using different scales. Risk ratios or odds ratios with 95% CI were applied to represent results for dichotomous outcomes. Statistical heterogeneity across the included studies was tested using the χ^2^ test and I^2^ statistics. Heterogeneity was considered statistically significant when the *p*-value based on the χ^2^ test was <0.10 or I^2^ was ≥50%. If heterogeneity was identified, a subgroup analysis was performed to explore the possible causes. Statistical synthesis of individual research results was performed using R version 4.1.0, with the default settings of the ‘meta’ package and the ‘metaprop’ function [29]. Only the random effects model was adopted in this review to statistically examine the results conservatively. To distinguish publication bias, a contour-enhanced funnel plot was used for the outcome, which included most of the studies [30]. For the asymmetry on the visually confirmed funnel plot, Egger’s test and Begg’s test were additionally performed to specifically confirm the existence of publication bias.

#### 2.5.2. Association Rule Analysis

By analyzing the constituent herb data of EAHM collected from the included studies, the potential association rules of core herb combinations were explored. Furthermore, prior to the association rule analysis, the frequency of individual herbs used in this analysis was checked. The R studio program (version 1.4.1106; Integrated Development for R. RStudio, PBC; Boston, MA, USA) was used for the Apriori association rule analysis and plot production. A data fit was performed using the R-package “arules” and the R-package “arulesViz”was applied to generate plots and charts according to the results [31,32]. The association rule analysis according to the Apriori algorithm is a data mining method for discovering meaningful correlations between two or more components included in one event [33]. This identifies the elements that compose the data and the relationship between the elements, and is used in various types of medical research aimed at predicting the variable characteristics [34,35,36]. This analysis does not identify a separate cause and aims to derive a rule from a combination of characteristics without a target variable.

Support, confidence, and lift are the main metrics used to measure associations using the Apriori algorithm. The metric support evaluates the usefulness of the association rule and is the proportion of prescriptions containing a specific herb combination in the total EAHM prescription. This can be expressed as P(A∩B). The metric confidence indicates the likelihood that the consequent herb set will be included when an antecedent herb set is specified as an EAHM prescription. Therefore, support is the entire set of standard EAHM prescriptions, whereas confidence limits reference prescriptions to those that include a specific herb combination and is expressed as P(A∩B)/P(A) = P(B|A). The metric lift compensates for the fact that it is not known whether confidence is useful or a random result. The confidence of herbs A and B is divided by the confidence under the independent assumption that A does not affect B, and is expressed as P(A∩B)/P(A)·P(B) = P(B|A)/P(B). When the confidence is approximately 1, herbs A and B are considered irrelevant since they are close to independence in probability. Conversely, if the lift value is large, the correlation is interpreted as strong. In this review, the association rules were identified based on the minimum values for support and confidence of 20 and 80%, respectively. Among them, the core herb combination showing the most distinct association and its constituent herbs was searched.

### 2.6. Quality of Evidence According to Outcome Measurements

The overall quality of evidence for each outcome was evaluated using the Grading of Recommendations Assessment, Development, and Evaluation (GRADE) pro [37]. The GRADE assessment evaluates the overall quality of evidence in four levels: Very low, low, moderate, and high. The level of evidence is degraded according to factors, such as risk of bias, inconsistency, indirectness, imprecision, and publication bias.

## 3. Results

### 3.1. Study Selection

**Figure 1:**
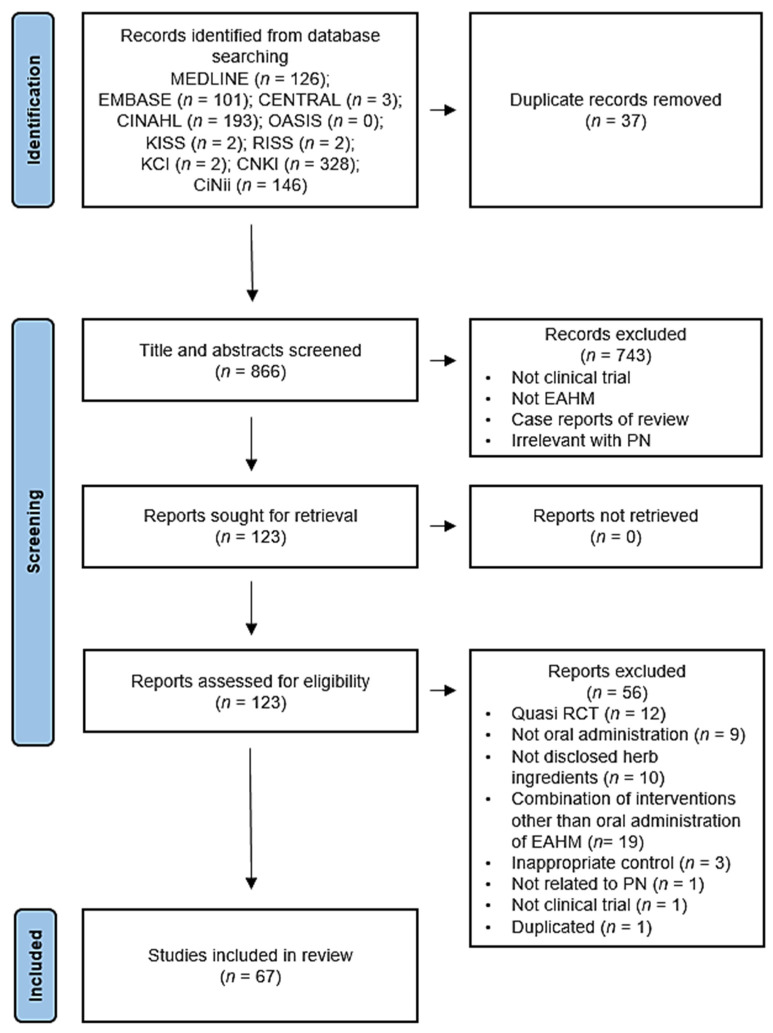
PRISMA 2020 flow diagram.

A total of 903 studies were selected through an electronic database search, of which 37 duplicates were removed. After screening for titles and abstracts, 743 studies were excluded for at least one of the following reasons: (i) No clinical trial, (ii) studies unrelated to EAHM, (iii) case reports or reviews, and (iv) irrelevant to PN. A full text assessment was performed on the remaining 123 studies, and 56 studies were excluded for the following reasons: (i) No clinical trials or quasi-RCTs; (ii) no oral administration; (iii) undisclosed herb ingredients; (iv) combination of interventions other than oral administration of EAHM; (v) inappropriate control groups; (vi) not related to PN; (vii) duplicated. A total of 67 studies were identified. The screening process is summarized in Figure 1.

### 3.2. Study Characteristics

Four RCTs were published in English, and the rest were published in Chinese. Four studies were conducted in Japan, whereas the others were conducted in China. The etiology of PN included studies of 50 DPNs, 11 CIPNs, one HPN, one occipital neuralgia, one trigeminal neuralgia, and one supraorbital neuralgia. The sample size of the included studies ranged from 29 to 247, and a total of 5753 participants were separated into the experimental group (*n* = 2898) and the control group (*n* = 2855). The treatment duration ranged from 2 to 26 weeks. The characteristics of the included 67 studies are summarized in Table 1.

### 3.3. Risk of Bias

**Figure 2:**
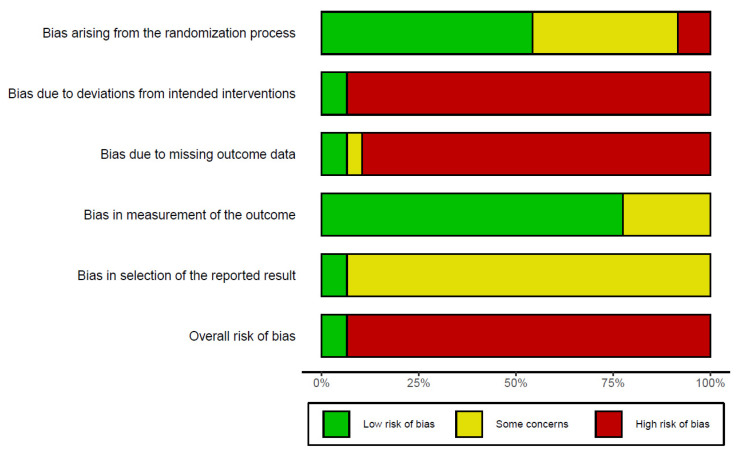
Risk of bias 2.0 graph of the included studies.

The methodological quality of the 67 included studies is summarized in Table 2 and Figure 2. The risk of bias was assessed using the RoB 2.0 tool [26]. Four studies were assessed to have a “low risk of bias” and the remaining 63 studies were assessed to have a ‘high risk of bias’.

### 3.4. Efficacy

#### 3.4.1. Primary Outcome: Sensory NCV (SNCV)

**Figure 3:**
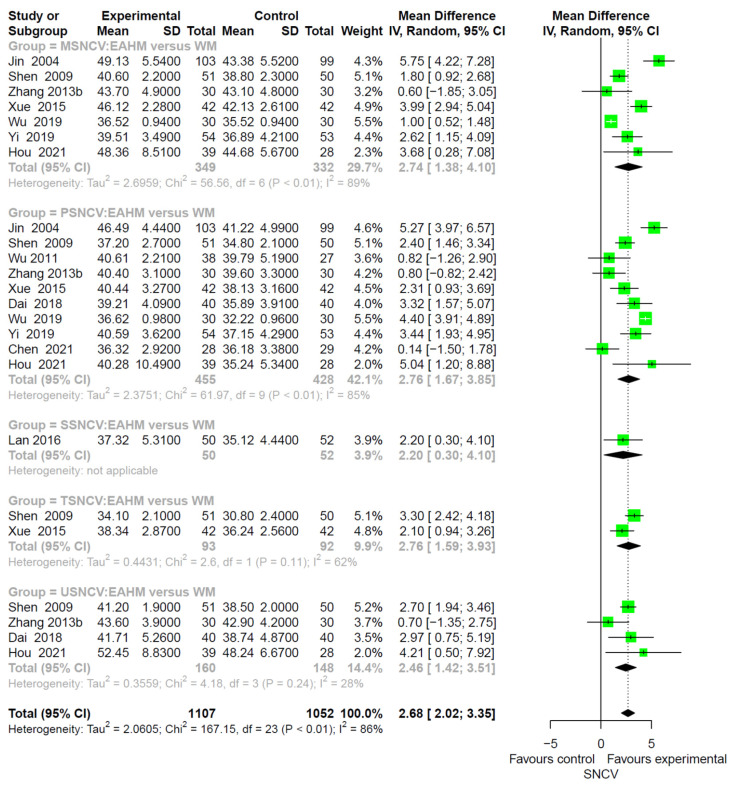
Forest plot of the trials reporting the effect of East Asian herbal medicine monotherapy on sensory nerve conduction velocity for peripheral neuropathy.

SNCV was measured in 31 studies, including 10 studies on EAHM monotherapy [38,40,44,49,55,59,70,77,81,82] and 21 studies on combined EAHM and western medicine (WM) therapy [41,42,45,46,48,53,57,63,64,65,67,68,71,72,75,79,84,86,87,95,96]. The studies on EAHM monotherapy compared the effect of EAHM on SNCV with WM. The combined effect of EAHM monotherapy was significantly better than the WM control (*n* = 2159; MD 2.68, 95% CI 2.02–3.35, *p* < 0.0001; heterogeneity chi-square = 167.15, df = 23, *p* < 0.01; I^2^ = 86; Figure 3).

**Figure 4:**
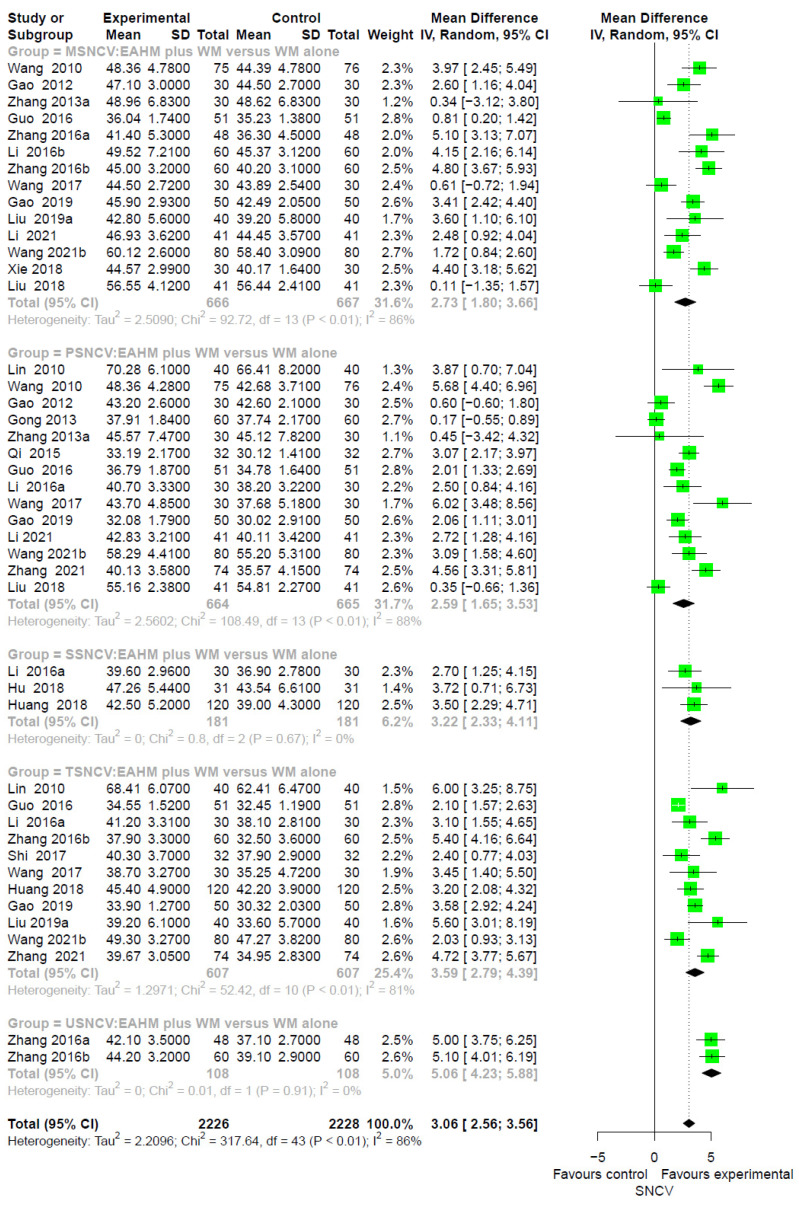
Forest plot of the trials reporting the effect of combined East Asian herbal medicine and western medicine therapy on sensory nerve conduction velocity for peripheral neuropathy.

In the 21 studies comparing the effect of combined EAHM and WM therapy with the WM monotherapy control, the combined therapy significantly improved SNCV than the WM monotherapy control (*n* = 4454; MD 3.06, 95% CI 2.56–3.56, *p* < 0.0001; heterogeneity chi-square = 317.64, df = 43, *p* < 0.01; I^2^ = 86%; Figure 4).

#### 3.4.2. Primary Outcome: Motor NCV (MNCV)

**Figure 5:**
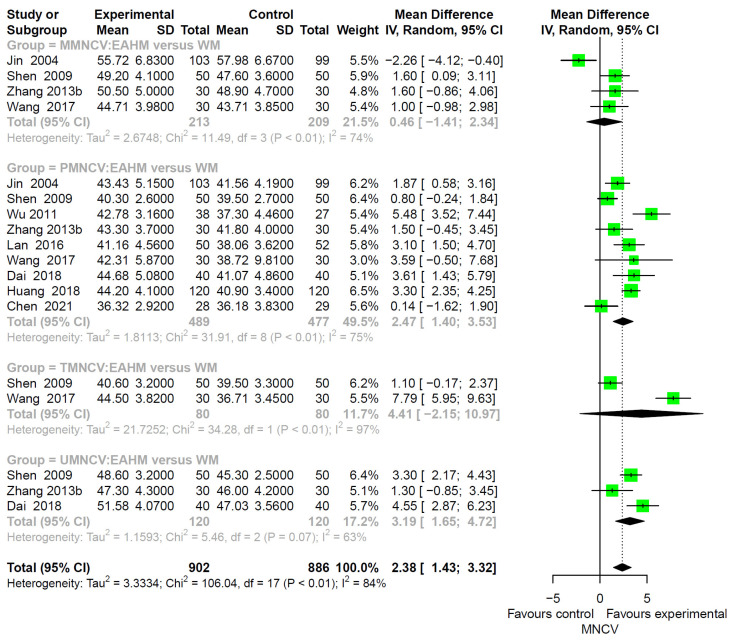
Forest plot of the trials reporting the effect of East Asian herbal medicine monotherapy on motor nerve conduction velocity for peripheral neuropathy.

MNCV was measured in 25 studies, including nine studies on EAHM monotherapy [38,40,44,49,59,68,70,72,81] and 16 studies on combined EAHM and WM therapy [41,42,45,46,48,53,57,63,65,71,77,79,84,86,95,96]. The combined effect of EAHM monotherapy on MNCV was significantly higher than the WM control (*n* = 1788, MD 2.38, 95% CI 1.43–3.32, *p* < 0.0001; heterogeneity chi-square = 179.27, df = 17, *p* < 0.01; I^2^ = 84%; Figure 5).

**Figure 6:**
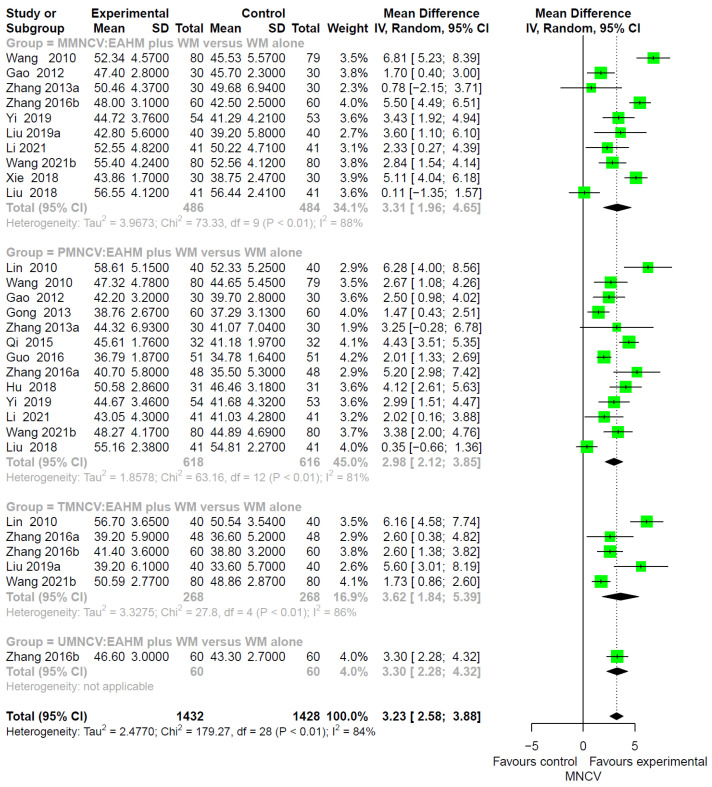
Forest plot of the trials reporting the effect of combined East Asian herbal medicine and western medicine therapy on motor nerve conduction velocity for peripheral neuropathy.

In addition, the combined EAHM and WM therapy significantly improved MNCV than WM monotherapy (*n* = 2860, MD 3.23, 95% CI 2.58–3.88, *p* < 0.0001; heterogeneity chi-square = 179.27, df = 28, *p* < 0.01; I^2^ = 84%; Figure 6).

#### 3.4.3. Primary Outcome: Response Rate

**Figure 7:**
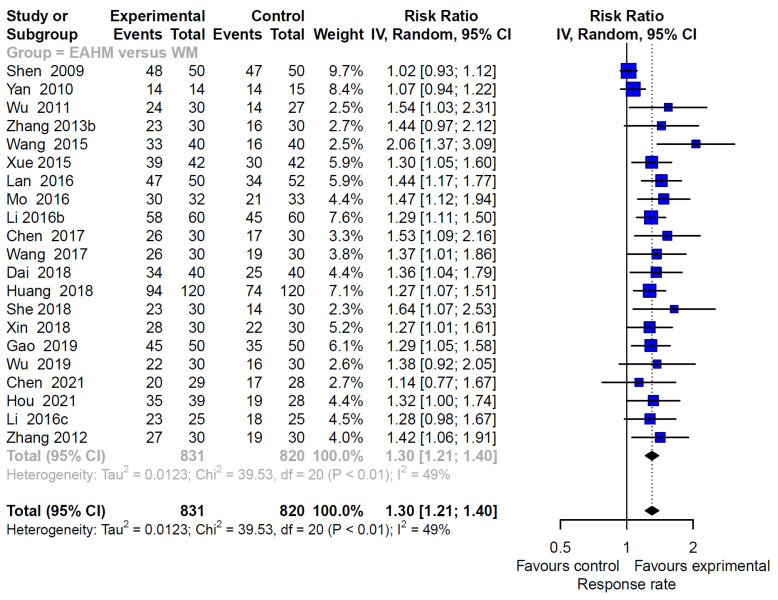
Forest plot of the trials reporting the effect of East Asian herbal medicine monotherapy on the response rate for peripheral neuropathy.

**Figure 8:**
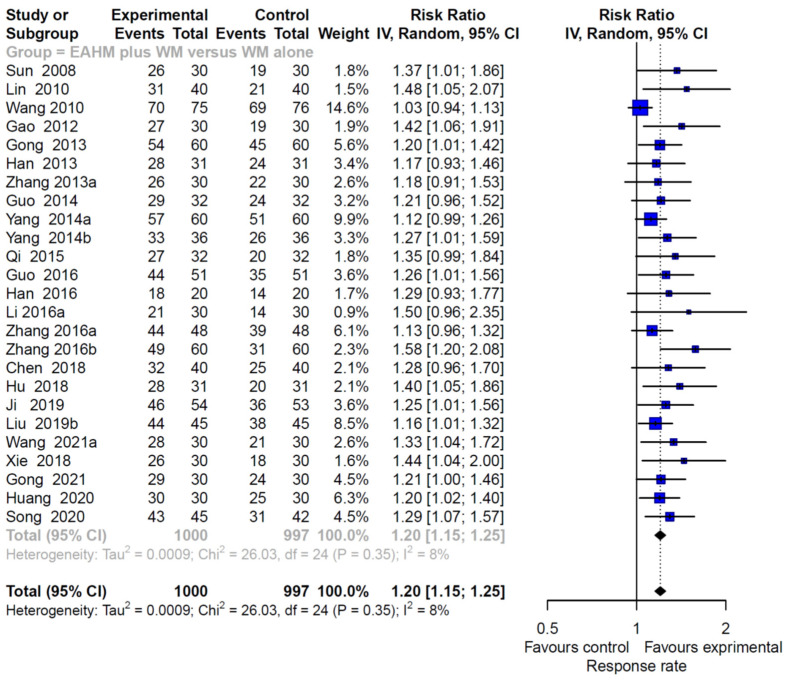
Forest plot of the trials reporting the effect of combined East Asian herbal medicine and western medicine therapy on the response rate for peripheral neuropathy.

**Figure 9:**
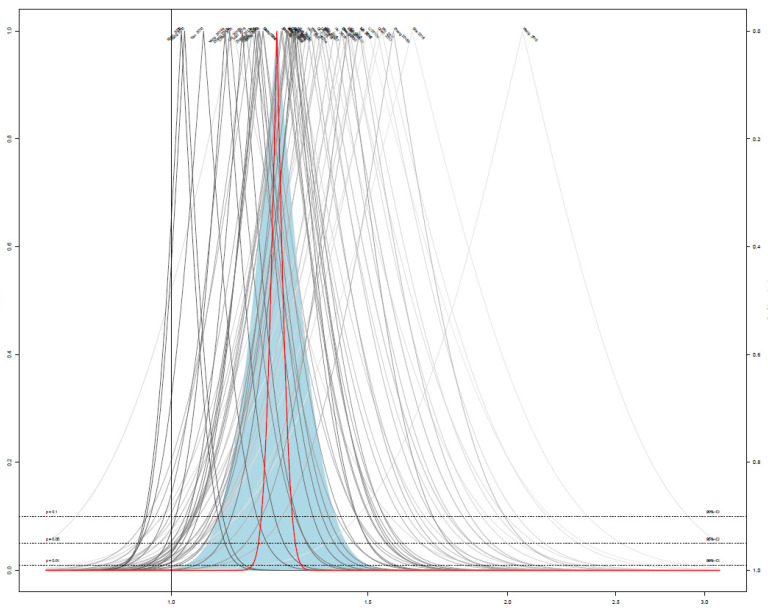
Drapery plot of the trials reporting the effect of East Asian herbal medicine monotherapy on the response rate for peripheral neuropathy.

The response rate was assessed in 48 studies, including 22 studies on EAHM monotherapy [40,43,44,49,54,55,59,60,64,66,68,70,72,73,74,75,76,81,82,99,100] and 26 studies on combined EAHM and WM therapy [39,41,42,45,46,47,48,50,51,52,53,57,58,61,62,63,65,69,71,78,80,85,95,102,103,104]. Twenty-five studies [39,41,42,45,46,47,48,50,58,62,63,65,69,71,78,80,85,95,103,104] compared the effect of EAHM monotherapy on the response rate with WM, and the remaining study [61] compared it with the untreated control. The combined effect of EAHM monotherapy on the response rate was significantly better than the WM control (*n* = 1651, risk ratio (RR) 1.30, 95% CI 1.21–1.40, *p* < 0.0001; heterogeneity chi-square = 39.53, df = 20, *p* < 0.01; I^2^ = 49%; Figure 7). Additionally, the combined EAHM and WM therapy significantly improved the response rate than the WM monotherapy (*n* = 1997, RR 1.20, 95% CI 1.15–1.25, *p* < 0.0001; heterogeneity chi-square = 26.03, df = 24, *p* = 0.35; I^2^ = 8%; Figure 8). The effect on the response rate was also significant in one study comparing EAHM monotherapy with the untreated control (*n* = 227, RR 1.19, 95% CI 1.03–1.37, *p* < 0.01). A visual summary of the confidence level for individual studies and pooled estimates using the response rate is presented through a drapery plot (Figure 9).

#### 3.4.4. Secondary Outcome: Incidence Rate

**Figure 10:**
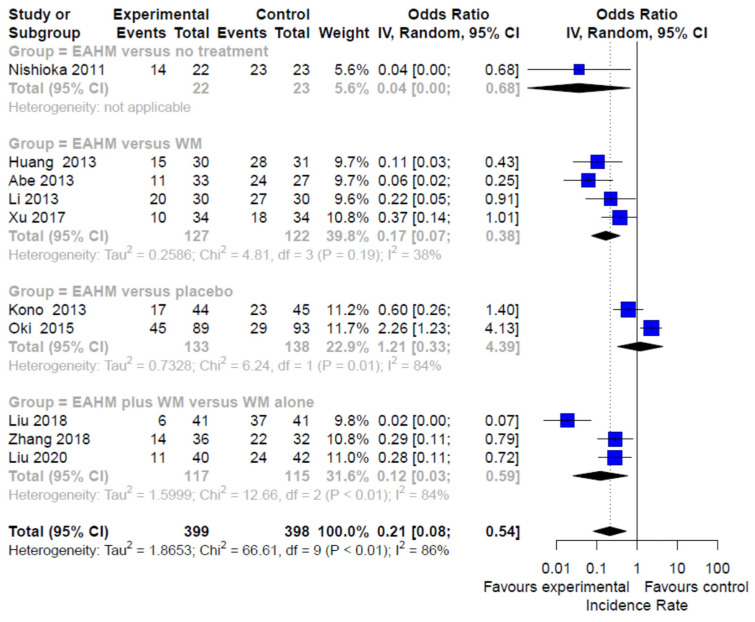
Forest plot of the trials reporting the effect of East Asian herbal medicine monotherapy on the incidence rate for peripheral neuropathy.

The incidence rate was reported in 11 studies [88,89,90,91,92,93,94,96,97,98]. Compared with no treatment, the odds of the incidence rate were significantly lower in the EAHM monotherapy group (one trial, *n* = 45, OR 0.04, 95% CI 0.00–0.68, *p* < 0.0001, Figure 10). In addition, the odds of the incidence rate in the EAHM monotherapy group were significantly lower than that in the WM group (four trials, *n* = 249, OR 0.17, 95% CI 0.07–0.38, *p* < 0.0001; heterogeneity chi-square = 4.81, df = 3, *p* = 0.19; I^2^ = 38%; Figure 10). The incidence rate in the combined EAHM and WM therapy group was also significantly lower than the WM monotherapy group (three trials, *n* = 232, OR 0.12, 95% CI 0.03–0.59, *p* < 0.0001; heterogeneity chi-square = 12.66, df = 2, *p* < 0.01; I^2^ = 84%; Figure 10). However, there was no significant difference in the odds of incidence rate between the EAHM monotherapy group and the placebo group (two trials, *n* = 271, OR 1.21, 95% CI 0.33–4.39, *p* = 0.7763; heterogeneity chi-square = 6.24, df = 1, *p* = 0.01; I^2^ = 84%; Figure 10).

#### 3.4.5. Secondary Outcome: Pain Intensity

**Figure 11:**
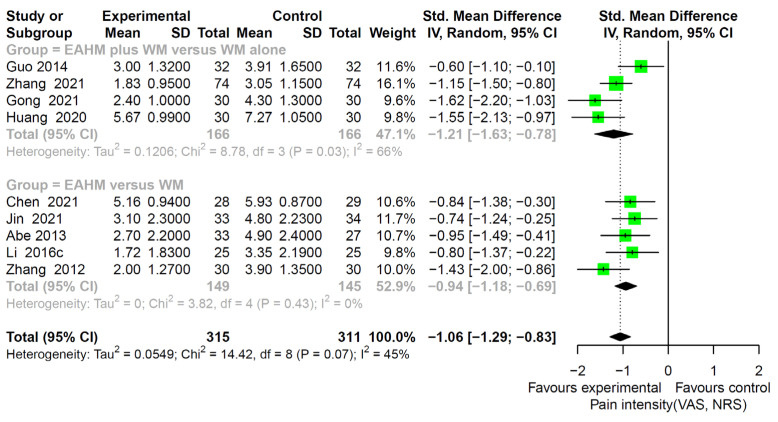
Forest plot of the trials reporting the effect of East Asian herbal medicine monotherapy on pain intensity for peripheral neuropathy.

Pain intensity was reported in nine studies [50,81,83,87,90,99,100,102,103]. The reduction in pain intensity was significantly greater in the EAHM monotherapy group than the WM monotherapy group (five trials, *n* = 294, SMD −0.94, 95% CI −1.18–−0.69, *p* < 0.0001; heterogeneity chi-square = 8.78, df = 3, *p* = 0.07; I^2^ = 45%; Figure 11). Compared with the WM monotherapy group, the meta-analysis showed a significantly lower effect of combined EAHM and WM therapy (four trials, *n* = 232, SMD −1.21, 95% CI −1.63–−0.78, *p* < 0.0001; heterogeneity chi-square = 8.78, df = 3, *p* = 0.03; I^2^ = 66%; Figure 11).

#### 3.4.6. Secondary Outcome: TCSS

**Figure 12:**
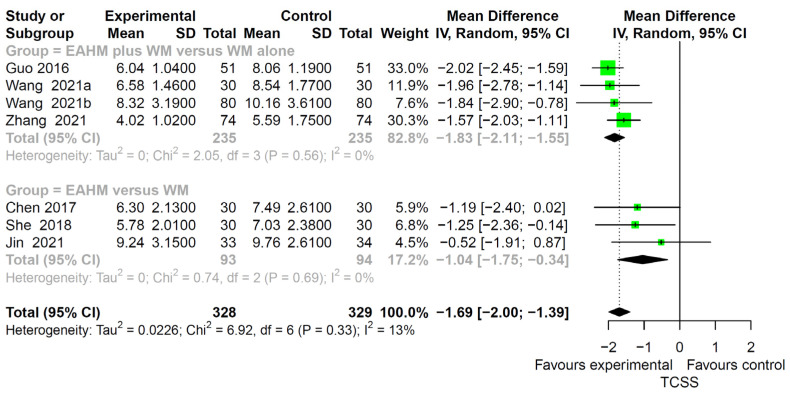
Forest plot of the trials reporting the effect of East Asian herbal medicine monotherapy on the Toronto clinical scoring system (TCSS) for peripheral neuropathy.

The effect of EAHM on the TCSS was described in seven studies [57,66,73,83,85,86,87]. A significant improvement in TCSS by EAHM monotherapy was identified by the WM monotherapy (three trials, *n* = 187, MD 1.04, *p* < 0.0001; heterogeneity chi-square = 0.74, df = 2, *p* = 0.69; I^2^ = 0%; Figure 12). Compared with WM monotherapy, the combined EAHM and WM therapy also showed a significantly lower effect on TCSS (four trials; *n* = 470, MD −1.83, *p* < 0.0001; heterogeneity chi-square = 2.05, df = 3, *p* = 0.69; I^2^ = 0%, Figure 12).

#### 3.4.7. Secondary Outcome: MDNS

**Figure 13:**
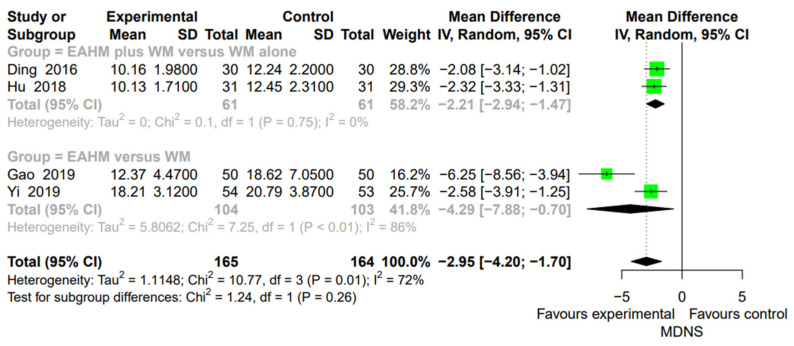
Forest plot of the trials reporting the effect of East Asian herbal medicine monotherapy on the Michigan diabetic neuropathy score (MDNS) for peripheral neuropathy.

The effect of EAHM on the MDNS was proven in four studies [56,71,75,77]. The meta-analysis revealed a significant reduction in MDNS by EAHM monotherapy (two trials, *n* = 207, MD 4.29, *p* < 0.0001; heterogeneity chi-square = 7.25, df = 1, *p* < 0.01; I^2^ = 86%; Figure 13). Compared with WM monotherapy, the combined EAHM and WM therapy also showed a significantly lower effect on MDNS (two trials, *n* = 122, MD −2.21, *p* < 0.0001; heterogeneity chi-square = 0.1, df = 1, *p* = 0.75; I^2^ = 0%; Figure 13).

### 3.5. AEs

Of the total 67 studies included in this review, 26 studies reported adverse event monitoring [38,40,45,46,47,49,53,54,55,59,61,62,65,69,72,75,76,77,84,88,90,91,92,93,100,101]. Among these, nine studies [38,45,49,65,69,72,77,84,101] reported multiple AEs possibly related to EAHM, and five studies [88,90,91,92,93] reported AEs unrelated to EAHM. No AEs were observed in the 12 studies [40,46,47,53,54,55,59,61,62,75,76,100]. The number of patients with AEs was 28/1322 (2.12%) in the experimental group and 31/1296 (2.4%) in the control group. Seven studies (seven in experimental groups and six in control groups) reported that the most frequent AEs were gastrointestinal symptoms, including abdominal pain, diarrhea, abdominal bloating, nausea, vomiting, anorexia, xerostomia, diarrhea, and constipation [38,45,69,72,77,84,101]. Skin rash was reported as an adverse event related to the integumentary system in two studies [49,69] (one in experimental group and two in control groups). Dizziness was reported as an adverse event related to the nervous system in three studies [65,84,101] (two in experimental groups and three in control groups). In all of the included studies, no severe AEs, which were life-threatening or required treatment for a long period of time, were reported. The details of the AEs reported in each study are presented in Table 1.

### 3.6. Subgroup Analysis

Table 3 summarizes the results of subgroup analysis based on individual causative diseases of PN and NCV for each site measured in five or more studies. There were no substantial changes in the results of the subgroup analysis.

### 3.7. Further Analysis of EAHM Intervention

#### 3.7.1. EAHM Composition Distribution

A total of 156 herbs were prescribed in the 67 studies included in this review. The cumulative use frequency of the top 10 herbs was 40%. The list of herbs constituting the EAHM used for each study is separately organized in a Supplementary File (Supplementary Material S3). The top 10 most frequently prescribed herbs for PN were Astragali Radix, Angelicae Gigantis Radix, Paeoniae Radix, Cnidii Rhizoma, Cinnamomi Ramulus, Spatholobi Caulis, Achyranthis Radix, Glycyrrhyziae Radix et Rhizoma, Salviae Militorthizae Radix. The frequency distributions of the herbs are shown in Table 4.

#### 3.7.2. Apriori Algorithm-Based Association Rule Analysis

Nine association rules were identified in the analysis based on the composition of the 67 EAHM prescriptions included in this study (Table 5).

**Figure 14:**
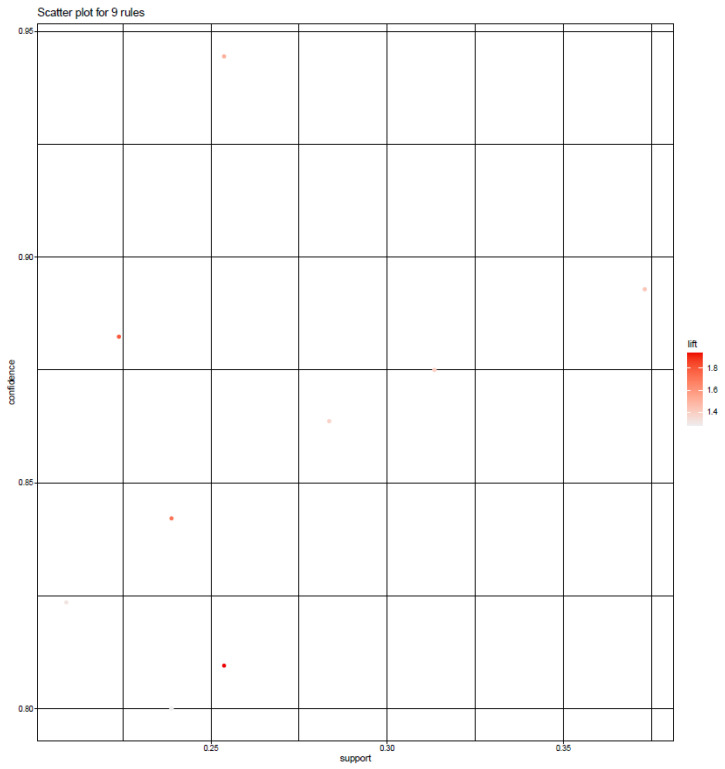
Scatter plot of the association rules in the meta-analysis of EAHM prescribed for peripheral neuropathy.

Subsequently, the distribution of the lift value was recognized through a scatter plot consisting of the association rule, with the support value on the *x*-axis and the confidence value on the *y*-axis (Figure 14).

**Figure 15:**
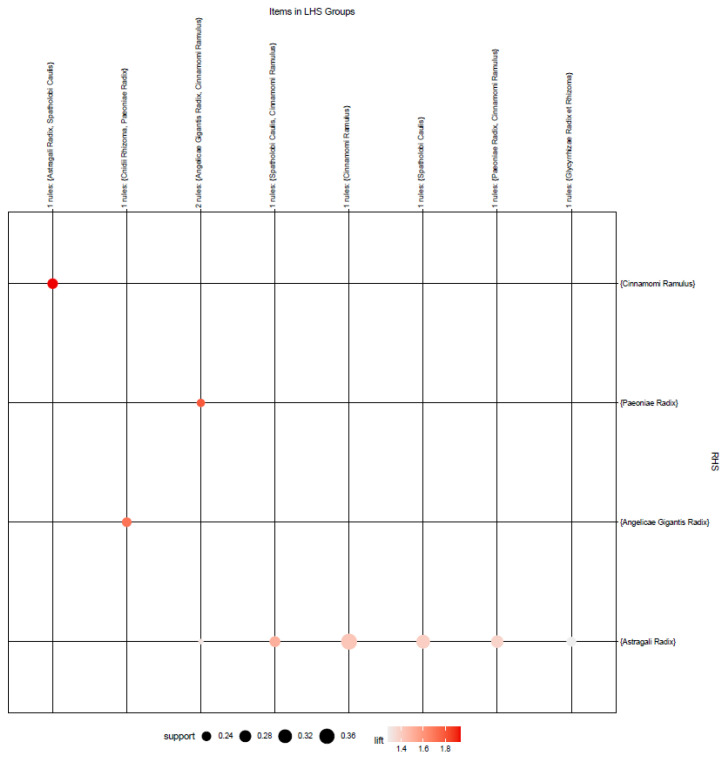
Grouping matrix of the association rules in the meta-analysis of East Asian herbal medicine prescribed for peripheral neuropathy.

The color depth of each association rule, determined by its lift value, confirmed that the distribution of the overall lift value ranged from 1.276 to 1.937. Meanwhile, a grouping matrix diagram was presented to examine the overall distribution of the identified association rule (Figure 15).

**Figure 16:**
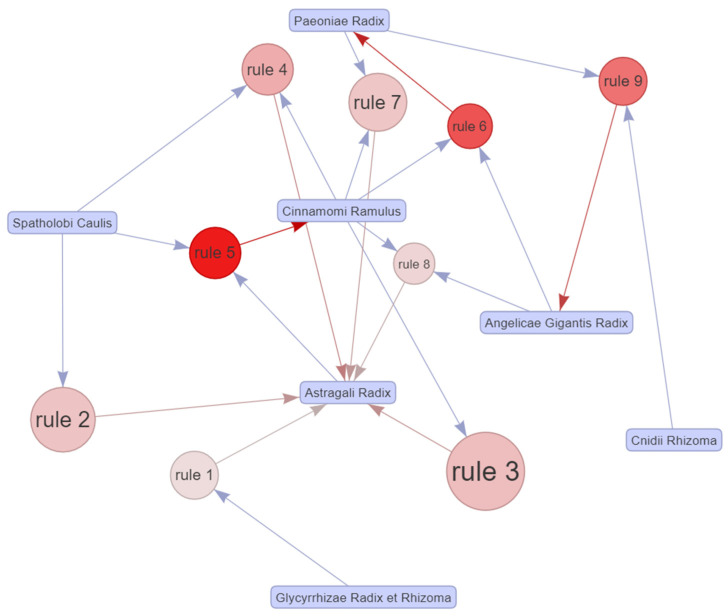
Network graph of the association rules in the meta-analysis of East Asian herbal medicine prescribed for peripheral neuropathy.

The horizontal ordinate shows eight association rules, and the vertical ordinate shows the items created by the eight rules. In this diagram, the depth of the color inside the circle represents the degree of lift, and the circle size represents the degree of support. From Figure 14 and Figure 15, the association rules of #2 {Spathologi Caulis} => {Astragali Radix}, #3 {Spathologi Caulis} => {Astragali Radix}, #4 {Astragali Radix, Spatholobi Caulis} => {Cinnamomi Ramulus}, and #5 {Astragali Radix, Spatholobi Caulis} => {Cinnamomi Ramulus} relevance can be identified. Looking at the specific value, there were two association rules with support exceeding 0.3, {Spatholobi Caulis} => {Astragali Radix} and {Spatholobi Caulis} => {Astragali Radix}. On the contrary, the only association rule indicating a confidence exceeding 0.9 was {Cinnamomi Ramulus, Spatholobi Caulis} => {Astragali Radix}. The association rule with the highest lift is {Astragali Radix, Spatholobi Caulis} => {Cinnamomi Ramulus}. Therefore, the constituents of the herb combinations with consistent association rules were Astragali Radix, Cinnamomi Ramulus, and Spatholobi Calulis. The relationship between these association rules is presented through a network graph (Figure 16).

### 3.8. Publication Bias

**Figure 17:**
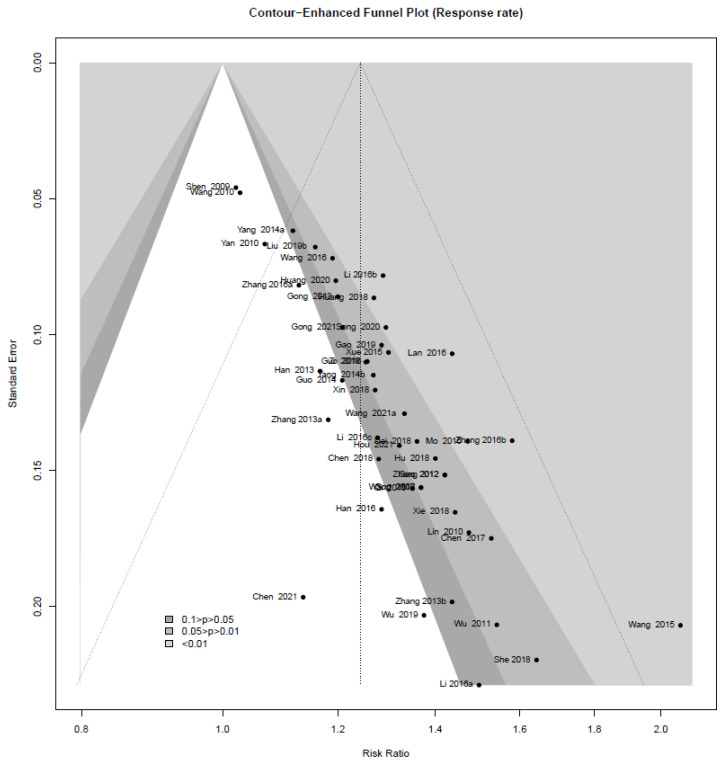
Contour-enhanced funnel plot for the meta-analysis of East Asian herbal medicine prescribed for peripheral neuropathy.

The contour-enhanced funnel plot analysis was performed to explore the publication bias through the response rate, which is an outcome of most of the included studies (Figure 17). The pattern of the funnel plot with 47 studies shows a clear asymmetry, indicating that there might have been publication bias (Figure 11). This was further confirmed by Egger’s test (*p* < 0.0001) and Begg’s test (*p* < 0.0001).

### 3.9. Quality of Evidence According to the Outcome Measurements

In the comparison between the combination EAHM and WM therapy and WM monotherapy, the overall quality of evidence according to all of the outcome measures was low to moderate. Meanwhile, the overall quality of evidence according to all of the outcome measures was low to moderate in EAHM monotherapy compared with WM monotherapy. The results of the GRADE assessment are listed in Table 6.

## 4. Discussion

### 4.1. Summary of the Main Finding

In this systematic review, 67 RCTs including 5753 PN patients were obtained and analyzed. The main finding of this study is that EAHM monotherapy or combined EAHM and WM therapy was superior to the control group without EAHM in improving nerve conduction velocity, response rate, incidence rate, pain intensity, and other overall symptoms. Additionally, EAHM is generally safe and tolerable for PN patients. Therefore, EAHM can be considered a recommended option for PN treatment in clinical practice based on the evidence presented in this study. On the contrary, in the association rule analysis of various EAHM prescription data included in this study, Astragali Radix, Cinnamomi Ramulus, and Spatholobi Calulis were identified as components constituting the core herb combination. It may be worthwhile to conduct further studies on whether EAHM containing the three individual herbs or their combination can exert a remarkable effect in the PN-treated group.

### 4.2. Limitations

This review has various limitations. Therefore, caution is required before using the results. First, most of the studies were conducted in China. As a result, additional well-designed multicenter clinical trials in East Asia are needed to generalize the positive results identified by the analysis. Second, the methodological quality of the clinical trials included in this study was generally poor. The overall risk of evaluated bias according to RoB 2.0 reported that only four included studies have a ‘low risk of bias.’ All of the other studies have a ‘high risk of bias’ due to methodological flaws in domains, such as the randomization process, deviations from the intended intervention, and missing outcome data. Therefore, it is difficult to draw firm conclusions, even though the review contains relatively large sample data and primary trials. Rigorous conclusions regarding EAHM can be drawn only in well-designed clinical trials to minimize the risk of future bias. Third, a high level of heterogeneity was observed in the meta-analysis of NCV, which is one of the primary outcomes of this study. This high heterogeneity is a problem that cannot be overlooked, as it reduces the significance of the synthesized evidence. In this study, the cause of heterogeneity could not be identified, even though a subgroup analysis was performed according to the underlying disease and treatment duration. It is estimated that the cause of the estimated troubleshooting is that the NCV at the basement and the amount of change in participants are different for each included study. This reflects the difficulty in diagnosing and measuring PN severity. Another possible cause of heterogeneity is the extreme diversity in the composition and dose of EAHMs used in individual clinical trials. This leads to inconsistency among interventions, except for the commonality of ‘the combination of herbal medicines in East Asia.’ In this review, the association rule analysis was performed on herb data to overcome this heterogeneity and derive useful information. The potential heterogeneity may be partially overcome in similar future systematic reviews by actively utilizing data mining methods. Fourth, the goal of this study was to identify valuable candidates for drug discovery or locate material information that may be employed in direct patient treatment in the clinic. Therefore, it was not possible to focus on quality control in the manufacturing process, such as pre-treatment, active ingredient extraction methods, and moisture content assessment, all of which significantly impact the efficacy of specific goods. Moreover, this was suspected to have influenced the heterogeneity of the results. In the future, an animal study meta-analysis on the same issue will be used to compensate for these flaws.

### 4.3. Implications of Clinical Practices

The evidence related to the use of EAHM therapy for PN supported by this study is consistent with the results of previous studies on similar subjects. A study analyzing the clinical data for DPN after using 216 EAHM prescriptions found that the combination of Astragali Radix-Cinnamomi Ramulus and Ligusticum Chuanxiong-Moutan Radicis Cortex highly correlated with MDNS improvement [105]. Moreover, considering that Astragali Radix and Cinnamoni Ramulus are among the top 10 herbs utilized and their combination was identified as a core herb pattern, the findings of the previous study are similar to this study. A systematic review in 2016 evaluating the effectiveness of EAHM formulation containing Astragali Radix as a central component for CIPN, also demonstrated a significant effect on the effective percentage and NCV [13]. Unlike this review, which dealt only with oral administration, this study elucidates the effects of topical preparation and injection. The differences between the two studies suggest that similar EAHMs can be effective when applied to PN even if they are administered through various routes. In 2020, a meta-analysis evaluating the effect of EAHM foot bath on DPN [106] was published, which also reported a significant improvement in SNCV, MNCV, and response rate in DPN patients after the EAHM treatment. However, the herbs frequently used in this study were Cinnamomi Ramulus, Carthami Flos, Herba Speranskiae Tuberculatae, and Cnidii Rhizoma, indicating that they are almost irrelevant to the frequently used herbs described in this review. According to the comprehensive evidence of this study and related topics to date, it is relatively clear that using various EAHM forms in clinical practice can be a meaningful treatment for PN patients. However, both the administration route and data from individual studies must be considered to identify whether a specific formulation or herb can be an effective choice. In addition, it cannot be concluded from only a few clinical studies. Therefore, further studies should focus on the possible mechanisms related to this topic.

### 4.4. Implications of the Research

The characteristic of EAHM to treat complex diseases by stimulating many networks of human interaction systems at the systematic level through a multi-targeted approach is being investigated [107]. Therefore, the multicomponent-derived EAHM exerts a synergistic effect between multiple compounds in the process of acting on multiple targets, resulting in efficacy with decreased toxicity and side effects [107,108]. Therefore, for efficient EAHM utilization, it is important to consider the synergistic combination of herbs rather than the primary mechanism of individual herbs. In this regard, the principle of prescription using botanical medicine called “Kun-shi-Choa-sa” has traditionally been used to combine two or more herbal medicines in East Asia, and recently, the simplest form of multi–herbal mixture, “herb-pair” is also studied [109]. As the associated case, the combination of Astragali Radix and Angelicae Gigantis Radix, which are mostly used, has been reported to improve axonal growth by primarily stimulating the neurotrophic signaling pathway against damage to the central nervous system [110]. The authors of this study argued that the combination of two drugs through a network pharmacology and a methodology could promote neurological recovery by inhibiting the expression of NogoA by triggering a multipath pathway. In another case, studies on the combination of Cinnamomi Ramulus and Glycyrrhyziae Radix et Rhizoma, the herbs frequently featured in this review, showed significant differences in pharmacokinetic parameters compared to the use of each single herb [111]. Based on this mechanism, high peak concentration, slow elimination, and great exposure were observed in Cinnamomi Ramulus and Glycyrrhyziae Radix et Rhizoma. According to several studies reviewed to date, appropriate herbal combinations are highly likely to produce excellent pharmacological and pharmacodynamic results. As a research hypothesis to develop efficient EAHM-based drugs for PN in the future, some core herb patterns identified by the association rule analysis in this review are meaningful.

Information on the pharmacological action of individual herbs is also important for achieving the above purpose. Research on various pharmacotherapeutic targets is required for an effective drug treatment for diseases in which the overall pathology, such as PN, is not fully understood. Combining this basic study with the action-related information of the individual active ingredient of EAHM will make it possible to clearly predict the direction of the synergistic effect expected from multiple herb combinations. Even for DPN, research on several molecular targets, including the polyol pathway, hexamine pathway, PKC signaling, oxidative stress, AGEs pathway, PARP pathway, MAPK pathway, NF-κB signaling, TNF-α signaling, and cyclooxygenase pathway is conducted [112]. In this review, the mechanisms by which major herbs induce PN pathology through various pathways are included. First, Astragali Radix downregulates the phosphorylation of heavy neurofilaments to prevent axonal damage and suppress pain hypersensitivity by reducing astrocytes and microglia scattered in the spinal cord and brain [113]. In another study, the mechanism by which APS protects against nerve damage is through miR-138 upregulation in rat neural stem cells [114]. Cinnamomi ramulus not only exerts neuroprotective effects by reducing oxidative damage and MDA and NO production, but also significantly suppresses pain hypersensitivity associated with inflammation [115,116]. Total glucosides in Paeoniae Radix protect against neurotoxicity, lower the level of neuronal nitric oxide synthase, and exhibit anti-nociceptive activity related to calcium channels [117,118]. Spatholobi Caulis demonstrated the therapeutic effects on neurological disorder-associated cell death by inhibiting JNK and p38 MAPK activation and reducing oxidative stress and apoptosis in a rat model of induced middle cerebral artery occlusion [119]. As mentioned above, studies related to the mechanism of action of herbs theoretically support the clinical effect of EAHM on PN, as confirmed in this review. However, in addition to these individual mechanisms, experimental studies are needed to identify targets that can reproducibly exert the synergistic effects of EAHM. Furthermore, future studies on whether the combined effects of EAHM actually produce clinical results distinguishable from the additive effects of individual agents, need to be performed.

### 4.5. Challenges and Perspectives

The following problems must be considered until the aforementioned discoveries are meaningfully exploited in clinical practice and medication discovery. Natural medicines, including EAHM and synthetic drugs, have significantly distinct modes of action, target pathways, and pharmacologically active components from a macroscopic point of view. The most well-known difference is that multiple compounds present in herbal medicine operate on many targets and single compound synthetic medications work on single targets [109]. As demonstrated in this review, most of the EAHM prescriptions comprise a blend of several components in specific amounts, frequently in a single formula. In this instance, each component alone frequently does not demonstrate several therapeutic actions, such as the entire combination. The pharmacological activity of EAHM is thought to be due to the synergetic action of several chemical components targeting multiple sites and the simultaneous action of multiple chemical components targeting a single site [120,121]. This is thought to be the most significant difference between synthetic medicines and EAHM. As a result of these EAHM characteristics, it has been difficult to discover possible indications and mechanisms in the past. In addition, there has been a belief that it is difficult to derive social and medical contributions as much as synthetic medications. Recent scientific research, on the contrary, has indicated that the combinatorial effect of mixed EAHM preparation can be particularly effective for complicated disorders, such as PN, autoimmune disease, degenerative disease, and cancer, which do not react well with single compound-based modern pharmaceutics [121,122,123,124]. As an example, recent research has demonstrated that EAHM can be used in large-scale public health emergencies, such as COVID-19 or preventive medicine using modern analytical tools, such as synthetic biology, data mining, and genomics [123,125,126,127,128]. Future studies need to be conducted to identify the properties of EAHM to be utilized in actual drug discovery.

First, this review was undertaken with the goal of finding EAHM materials that may provide prospective advantages to PN patients, and it does not go into detail regarding the formulation process of the materials. However, as mentioned above, estimating a consistent impact even for the same herb material in a condition where there is a lack of adequate consensus and discussion on the processing technique, pre-treatment, and extraction method of individual EAHM materials may be challenging [129,130]. Since most of the EAHM dosage forms discussed in this study are decoctions, it is also important to include various methods for determining the water content of the product [131]. These issues need to be addressed in a review of animal studies focusing on this subject. Simultaneously, it is thought that providing standardized information on the above in future EAHM-related clinical trials would help in enhancing the quality control of herbal materials. Second, while comparing EAHM to natural materials with efficacy against PN is outside the scope of this review, it is deemed necessary. For example, several clinical trials have gathered early data for Cannabis sativa. Moreover, additional materials, such as mulberry, Citrullus colocynthis, Matricia chamomilla, and Myristica fragrans have promising benefits for PN [9,132]. It is envisaged that relevant drug discovery information will be generated through a comparison of the phytochemical and clinical effectiveness with those of conventional herbal medicine in follow-up investigations.

## 5. Conclusions

This meta-analysis supports the hypothesis that EAHM monotherapy may be beneficial for PN patients. Moreover, the combined EAHM and WM therapy may be recommended for these patients. EAHM monotherapy improves severe pain intensity and abnormal sensations, such as tingling, burning, and numbness, which impair the quality of life in PN patients. Additionally, unlike the PN treatment with WM alone, which has a poor prognosis, a combination of EAHM and WM treatment alleviated the symptoms of PN including tingling, burning, and numbness and prevented chronic PN. However, high quality RCTs evaluating the effects of EAHM are needed due to limitations, such as heterogeneity, to understand this result clearly. In addition, it is worth conducting a follow-up study to verify the specific action target of the core herb combination derived from the present review and the hypothesis of superiority in clinical practice.

## Figures and Tables

**Table 1:** Characteristics of the included studies.

First Author (Year) [Reference]	Type of Condition	Trial Design	Number of Participants (Male/Female); Age (Mean ± SD)		Interventions		Morbidity Period (Mean ± SD or Range)		Outcome Index (Intergroup Differences *p*-Value)	Course of Treatment	Adverse Event (Case/Symptom)
			Trial	Control	Trial	Control	Trial	Control			
Jin (2004) [38]	DPN	RCT	103(54/49) 59.4 ± 5.61 y	99(51/48) 58.81 ± 6.01 y	Tangmaitong tablets (0.5 g × 4 t, t.i.d.)	Mecobalamin tablets (500 μg, t.i.d.)	3.31 ± 1.25 y	3.82 ± 1.17 y	1. MMNCV (*p* > 0.05) 2. MSNCV (*p* < 0.01) 3. PMNCV (*p* < 0.05) 4. PSNCV (*p* < 0.01)	8 w	Trial: 1 AE/diarrhea Control: 3 AEs/abdominal pain with diarrhea
Sun (2008) [39]	DPN	RCT	30(18/12) 40–70 y	30(16/14) 43–69 y	1. Ziyinbushenhuoxuetonglou fang decoction (300 mL, b.i.d.) 2. Mecobalamin tablets (500 μg, t.i.d.)	Mecobalamin tablets (500 μg, t.i.d.)	1–33 m	1–34 m	1. CER (*p* < 0.05)	4 w	NR
Shen (2009) [40]	DPN	RCT	50(21/29) 60 ± 4.2 y	50(27/23) 58.81 ± 6.01 y	Tangmaining capsule (4.5 g × 5 c, b.i.d.)	Mecobalamin tablets (500 μg, t.i.d.)	8.5 y	7.9 y	1. CER (*p* < 0.05) 2. MMNCV (*p* < 0.05) 3. MSNCV (*p* < 0.05) 4. UMNCV (*p* < 0.01) 5. USNCV (*p* < 0.01) 6. PMNCV (*p* < 0.05) 7. PSNCV (*p* > 0.05) 8. TMNCV (*p* > 0.05) 9. TSNCV (*p* < 0.01)	8 w	Trial: No AE Control: No AE
Lin (2010) [41]	DPN	RCT	40(22/18) median 55.6 y	40(23/19) median 54.2 y	1. Tongxinluo capsule (3 c, t.i.d.) 2. Mecobalamin tablets (500 μg, t.i.d.)	Mecobalamin tablets (500 μg, t.i.d.)	NR	NR	1. CER (*p* < 0.05) 2. PMNCV (*p* < 0.01) 3. PSNCV (*p* < 0.01) 4. TMNCV (*p* < 0.01) 5. TSNCV (*p* < 0.01)	4 w	NR
Wang (2010) [42]	DPN	RCT	80(45/35) 62.68 ± 7.35 y	79(43/36) 62.78 ± 7.57 y	1. Huangqiguizhiwuwu decoction (300 mL, b.i.d.) 2. Mecobalamin injection (0.5 mg, q.d., i.m.)	Mecobalamin injection (0.5 mg, q.d., i.m.)	7.12 ± 4.25 y	6.98 ± 4.62 y	1. CER (*p* < 0.01) 2. MMNCV (*p* < 0.01) 3. MNSCV (*p* < 0.01) 4. PMNCV (*p* < 0.01) 5. PSNCV (*p* < 0.01)	12 w	NR
Yan (2010) [43]	DPN	RCT	14(7/7) 57.79 ± 6.73 y	15(6/9) 52.53 ± 8.0 y	Shutangluofang granule (b.i.d.)	Methylcobalamine (500 μg, t.i.d.)	13.14 ± 10.58 m	10.67 ± 11.14 m	1. CER (*p* < 0.05)	12 w	NR
Wu (2011) [44]	DPN	RCT	30(16/14) mean 49.9 y	27(15/12) mean 48 y	Modified yiqihuoxue decoction (300 mL, b.i.d.)	Vitamin B1 (20 mg, t.i.d.) Vitamin B6 (20 mg, t.i.d.)	mean 12 m	mean 11.4 m	1. CER (*p* < 0.01) 2. PMNCV (*p* < 0.01) 3. PSNCV (*p* < 0.01)	6 w	NR
Gao (2012) [45]	DPN	RCT	30(16/14) NR	30(17/13) NR	1. Nourishing the liver to stop the wind and tongluo decoction 2. Methylcobalamine (0.5 mg, t.i.d.)	Methylcobalamine (0.5 mg, t.i.d.)	NR	NR	1. CER (*p* < 0.05) 2. MMNCV (*p* < 0.01) 3. MSNCV (*p* < 0.01) 4. PMNCV (*p* < 0.01) 5. PSNCV (*p* < 0.01)	8 w	Trial: 2 AEs/nausea, upper abdominal discomfort Control: No AE
Gong (2013) [46]	DPN	RCT	60(32/28) 56.42 ± 5.28 y	60(33/27) 57.16 ± 5.34 y	1. Modified aconite decoction (400 mL, b.i.d.) 2. Methylcobalamine (500 μg, t.i.d.)	Methylcobalamine (500 μg, t.i.d.)	7.65 ± 3.84 m	7.83 ± 3.29 m	1. CER (*p* < 0.05) 2. PMNCV (*p* < 0.01) 3. PSNCV (*p* > 0.05)	30 d	Trial: No AE Control: No AE
Han (2013) [47]	DPN	RCT	31(17/14) 54.2 ± 9.6 y	31(16/15) 55.3 ± 10.1 y	1. Modified huangqiguizhiwuwu decoction (400 mL, b.i.d.) 2. Methylcobalamine (0.5 mg, t.i.d.)	Methylcobalamine (0.5 mg, t.i.d.)	NR	NR	1. CER (*p* < 0.05)	8 w	Trial: No AE Control: No AE
Zhang (2013a) [48]	DPN	RCT	30(16/14) 54.32 ± 7.14 y	30(15/15) 56.24 ± 7.40 y	1. Mudan tong luo fang (b.i.d.) 2. α-Lipoic acid injection (600 mg, q.d., i.v. drip)	α-Lipoic acid injection (600 mg, q.d., i.v. drip)	8.3 ± 1.67 y	8.5 ± 1.54 y	1. CER (*p* < 0.05) 2. MMNCV (*p* < 0.05) 3. MSNCV (*p* < 0.05) 4. PMNCV (*p* < 0.05) 5. PSNCV (*p* < 0.05)	3 w	NR
Zhang (2013b) [49]	DPN	RCT	30 Total 60(36/14) 56 ± 8 y	30 Total 60(36/14) 56 ± 8 y	Tang bao kang (20 pills, t.i.d.)	1. Methylcobalamine (500 μg, t.i.d.) 2. Vitamin B1 (30 mg, t.i.d.) 3. Vitamin B6 (30 mg, t.i.d.)	Total 5–10 y	Total 5–10 y	1. CER (*p* < 0.01) 2. MMNCV (*p* < 0.01) 3. MSNCV (*p* < 0.01) 4. UMNCV (*p* < 0.01) 5. USNCV (*p* < 0.01) 6. PMNCV (*p* < 0.01) 7. PSNCV (*p* < 0.01)	24 w	Trial: No AE Control: 1 AE/skin rash
Guo (2014) [50]	DPN	RCT	32(19/13) 64.78 ± 8.90 y	32(15/17) 65.59 ± 8.35 y	1. Modified huangqiguizhiwuwu decoction (b.i.d.) 2. Mecobalamin tablets (0.5 mg, t.i.d.) 3. Gabapentin (600 mg, t.i.d.)	1. Mecobalamin tablets (0.5 mg, t.i.d.) 2. Gabapentin (600 mg, t.i.d.)	NR	NR	1. CER (*p* < 0.01) 2. VAS (*p* < 0.05)	8 w	NR
Yang (2014a) [51]	DPN	RCT	60(35/25) 51.30 ± 6.03 y	60(37/23) 51.26 ± 5.38 y	1. Shenqixuebi feng (b.i.d.) 2. α-Lipoic acid injection (0.3 g, q.d., i.v. drip) 3. Mecobalamin injection (0.5 mg, q.d., i.v. drip)	1. α-Lipoic acid injection (0.3 g, q.d., i.v. drip) 2. Mecobalamin injection (0.5 mg, q.d., i.v. drip)	3.65 ± 1.12 y	3.36 ± 1.18 y	1. CER (*p* < 0.05)	4 w	NR
Yang (2014b) [52]	DPN	RCT	36(23/13) 47.8 ± 8.3 y	36(20/16) 46.5 ± 8.1 y	1. Modified huangqiguizhiwuwu decoction (200 mL, q.d.) 2. Methylcobalamine injection (500 μg, q.d., i.m.)	1. Methylcobalamine injection (500 μg, q.d., i.m.)	4.1 ± 1.3 m	3.9 ± 1.4 m	1. CER (*p* < 0.05)	4 w	NR
Qi (2015) [53]	DPN	RCT	32(17/15) 53.2 ± 7.1 y	32(16/16) 52.4 ± 7.0 y	1. Mudan granule (7 g, t.i.d.) 2. 0.9% Sodium chloride 200 mL and α-Lipoic acid injection (450 mg, q.d., i.v. drip)	1. 0.9% Sodium chloride 200 mL and αLipoic acid injection (450 mg, q.d., i.v. drip)	2.3 ± 2.1 y	2.6 ± 1.9 y	1. CER (*p* < 0.05) 2. PMNCV (*p* < 0.01) 3. PSNCV (*p* < 0.01)	4 w	Trial: No AE Control: No AE
Wang (2015) [54]	DPN	RCT	40(20/20) mean 68.5 y	40(23/17) mean 71.2 y	1. Yinxinshu capsule (3 c, t.i.d.) 2. Maixuekang capsule (3 c, t.i.d.)	1. Oryzanol (20 mg, t.i.d.) 2. Vitamin B1 (10 mg, t.i.d.) 3. Adenosylcobalamin (1 mg, t.i.d.)	10–12 y	10–12 y	1. CER (*p* < 0.05)	4 w	Trial: No AE Control: No AE
Xue (2015) [55]	DPN	RCT	42(23/19) 36–78 y	42(22/20) 35–78 y	1. Modified liutengshuilushexian decoction (150 mL, q.d.)	1. Methylcobalamine tablet (0.5 mg, t.i.d.)	28–73 d	30–73 d	1. CER (*p* < 0.01) 2. MSNCV (*p* < 0.01) 3. TSNCV (*p* < 0.01) 4. PSNCV (*p* < 0.01)	3 w	Trial: No AE Control: No AE
Ding (2016) [56]	DPN	RCT	30(12/18) 55.16 ± 11.78 y	30(16/14) 54.97 ± 12.05 y	1. Buyanghuanwu decoction (b.i.d.) 2. Methylcobalamine (0.5 mg t.i.d.) 3. Alprostadil injection (10 ug, q.d., i.v.) 4. α-Lipoic acid injection (0.3 mg, q.d., i.v. drip)	1. Methylcobalamine (0.5 mg, t.i.d.) 2. Alprostadil injection (10 ug, q.d., i.v.) 3. α-Lipoic acid injection (0.3 mg, q.d., i.v. drip)	7.51 ± 2.12 y	6.59 ± 1.91 y	1. MDNS (*p* < 0.05)	8 w	NR
Guo (2016) [57]	DPN	RCT	51(26/25) 69.54 ± 5.06 y	51(28/23) 69.78 ± 5.96 y	1. Qitengtongluo decoction (b.i.d.) 2. Epalrestat (50 mg, 1 t, t.i.d.)	1. Epalrestat (50 mg, 1 t, t.i.d.)	1.91 ± 2.09 y	6.59 ± 1.91 y	1. CER (*p* < 0.05) 2. NCSS (*p* < 0.05) 3. MSNCV (*p* < 0.05) 4. TSNCV (*p* < 0.05) 5. PMNCV (*p* < 0.05) 6. PSNCV (*p* < 0.05)	12 w	NR
Han (2016) [58]	DPN	RCT	20(12/8) 54.3 ± 7.2 y	20(11/9) 53.7 ± 6.8 y	1. Zhanjin tongluo chinese medicine (b.i.d.) 2. Mecobalamin tablets (500 μg, t.i.d.)	1. Mecobalamin tablets (500 μg, t.i.d.)	2.4 ± 1.2 y	2.6 ± 1.3 y	1. CER (*p* < 0.05)	4 w	NR
Lan (2016) [59]	DPN	RCT	54 Other information NR	54 Other information NR	Yiqihuoxue tongluo capsule (1.2 g, t.i.d.)	Epalrestat tablets (50 mg, t.i.d.)	NR	NR	1. CER (*p* < 0.05) 2. PMNCV (*p* < 0.05) 3. SSNCV (*p* < 0.05)	12 w	Trial: No AE Control: No AE
Mo (2016) [60]	DPN	RCT	33(19/14) 65.28 ± 9.098 y	32(17/15) 62.34 ± 8.168 y	Yangyinjiedudecoction (300 mL, b.i.d.)	Methylcobalamine (0.5 mg t.i.d.)	2–23 y	2–19 y	1. CER (*p* < 0.01)	8 w	NR
Wang (2016) [61]	DPN	RCT	124(72/52) 57.3 ± 6.8 y	103(58/45) 58.1 ± 7.2 y	Modified tangbitong feng (150 mL, b.i.d.)	No treatment	22.1 ± 5.4 m	23.5 ± 4.8 m	1. CER (*p* < 0.01)	8 w	Trial: No AE Control: No AE
Li (2016a) [62]	DPN	RCT	30(18/12) 49.6 ± 5.6 y	30(17/13) 50.3 ± 5.4 y	1. Wenyanghuoxuetongbi feng (b.i.d.) 2. Methylcobalamine (0.5 mg, t.i.d.)	1. Methylcobalamine (0.5 mg, t.i.d.)	18.21 ± 12.37 m	17.97 ± 12.54 m	1. CER (*p* < 0.01) 2. TSNCV (*p* < 0.01) 3. SSNCV (*p* < 0.05) 4. PSNCV (*p* < 0.05)	8 w	Trial: No AE Control: No AE
Zhang (2016a) [63]	DPN	RCT	48(26/22) 54.6 y	48(28/20) 55.2 y	1. Huangichifeng decoction combined Dangguisini decoction (q.d.) 2. Methylcobalamine injection (500 μg, q.d., i.m.)	1. Methylcobalamine injection (500 μg, q.d., i.v.)	2.8 y	3.2 y	1. CER (*p* < 0.01) 2. MSNCV (*p* < 0.01) 3. USNCV (*p* < 0.01) 4. PMNCV (*p* < 0.01) 5. TMNCV (*p* < 0.01)	4 w	NR
Li (2016b) [64]	DPN	RCT	60(37/23) 57 y	60(35/25) 56 y	Huangzhitongnaoluo capsule (3 c, t.i.d.)	Mecobalamin dispersible tablets (500 mg, t.i.d.)	1–13 y	1–12 y	1. CER (*p* < 0.05) 2. MSNCV (*p* < 0.05) 3. TMNCV (*p* < 0.05)	12 w	NR
Zhang (2016b) [65]	DPN	RCT	60(36/24) 55.3 ± 6.4 y	60(35/25) 55.6 ± 5.5 y	1. Qiming granule (4.5 g, t.i.d.) 2. Nimodipine injection (8 mg, q.d., i.v. drip)	1. Nimodipine injection (8 mg, q.d., i.v. drip)	2.0 ± 1.1 y	2.2 ± 1.0 y	1. CER (*p* < 0.01) 2. MMNCV (*p* < 0.01) 3. MSNCV (*p* < 0.01) 4. UMNCV (*p* < 0.05) 5. USNCV (*p* < 0.01) 6. TMNCV (*p* < 0.05) 7. TSNCV (*p* < 0.01)	12 w	Trial: No AE Control: 1 AE/mild dizziness
Chen (2017) [66]	DPN	RCT	30(14/16) 38.72 ± 20.02 y	30(13/17) 39.11 ± 19.57 y	Dagguisini decoction (300 mL, b.i.d.)	Epalrestat capsule (50 mg, t.i.d.)	4.32 ± 2.05 y	4.20 ± 2.01 y	1. CER (*p* < 0.05) 2. TCSS (*p* < 0.05)	12 w	Trial: No AE Control: No AE
Shi (2017) [67]	DPN	RCT	32(20/12) 38.7 ± 8.1 y	32(22/10) 40.3 ± 10.1 y	Fufang danshen dripping pill (10 pills, t.i.d.)	1. Methylcobalamine (0.5 mg, t.i.d.) 2. Epalrestat (50 mg, t.i.d.)	3.87 ± 1.5 y	3.69 ± 1.3 y	1. TSNCV (*p* < 0.01)	15 w	NR
Wang (2017) [68]	DPN	RCT	30(15/15) 58.76 ± 4.32 y	30(16/14) 57.21 ± 3.56 y	Dangguisini decoction (200 mL, b.i.d.)	Mecobalamin tablets (500 μg, t.i.d.)	3.56 ± 1.21 y	3.84 ± 1.36 y	1. CER (*p* < 0.05) 2. MMNCV (*p* > 0.05) 3. MSNCV (*p* > 0.05) 4. PMNCV (*p* < 0.05) 5. PSNCV (*p* < 0.05) 6. TMNCV (*p* < 0.05) 7. TSNCV (*p* < 0.05)	8 w	NR
Chen (2018) [69]	DPN	RCT	40(19/21) 55.8 ± 4.7 y	40(20/20) 56.2 ± 2.8 y	1. Dangguisinin decoction (b.i.d.) 2. Mecobalamin tablets (500 μg, t.i.d.)	Mecobalamin tablets (500 μg, t.i.d.)	3.6 ± 1.8 y	2.4 ± 2.1 y	1. CER (*p* < 0.05)	4 w	Trial: 2 AEs/skin rash, gastrointestinal discomfort Control: 3 AEs/diarrhea (2), skin rash
Dai (2018) [70]	DPN	RCT	40 45–85 y Other information NR	40 45–85 y Other information NR	Modified huangqiguizhiwuwu decoction (500 mL, b.i.d.)	Epalrestat capsule (50 mg, t.i.d.)	NR	NR	1. CER (*p* < 0.05) 2. UMNCV (*p* < 0.05) 3. USNCV (*p* < 0.05) 4. PMNCV (*p* < 0.05) 5. PSNCV (*p* < 0.05)	3 w	NR
Hu (2018) [71]	DPN	RCT	31(13/18) 55.45 ± 11.52 y	31(15/16) 53.76 ± 2.03 y	1. Modified Jiajianhuangqiguizhiwuwu decoction (200 mL, b.i.d.) 2. Methylcobalamine (0.5 mg, t.i.d.)	1. Methylcobalamine tablet (0.5 mg, t.i.d.)	7.13 ± 2.01 y	6.52 ± 1.95 y	1. CER (*p* < 0.05) 2. SMNCV (*p* < 0.05) 3. SSNCV (*p* < 0.05) 4. MDNS (*p* < 0.05)	8 w	NR
Huang (2018) [72]	DPN	RCT	120(52/68) 51.3 ± 11.4 y	120(51/69) 50.9 ± 11.6 y	Matong powder (7 g, t.i.d.)	Methylcobalamine tablet (0.5 mg, t.i.d.)	8.92 ± 8.6 m	8.97 ± 8.5 m	1. CER (*p* < 0.05) 2. PMNCV (*p* < 0.05) 3. TSNCV (*p* < 0.05) 4. SSNCV (*p* < 0.05)	8 w	Trial: 3 AEs/ Abdominal bloating with anorexia (3) Control: 2 AEs/Abdominal bloating with anorexia (2)
She (2018) [73]	DPN	RCT	30(18/12) 63.35 ± 7.12 y	30(17/13) 65.13 ± 6.21 y	1. Huangqiguizhiwuwu granule (b.i.d.) 2. Mecobalamin tablet (1 mg, t.i.d.)	Mecobalamin tablet (1 mg, t.i.d.)	3.31 ± 2.06 y	3.82 ± 1.97 y	1. CER (*p* < 0.05) 2. TCSS (*p* < 0.05)	6 w	NR
Xin (2018) [74]	DPN	RCT	30 Total 60(36/24) 55.3 y	30 Total 60(36/24) 55.3 y	1. Mongolian medicine garidi-13 weiwan (3 g, q.d.)	Mecobalamin tablet (0.5 mg, t.i.d.)	Total 4.2 y	Total 4.2 y	1. CER (*p* < 0.05)	4 w	NR
Gao (2019) [75]	DPN	RCT	50(26/24) 60.83 ± 5.26 y	50(25/25) 61.17 ± 6.05 y	1. Modified shengmaisan (300 mL, b.i.d.) 2. Mecobalamin tablet (500 μg, t.i.d.)	Mecobalamin tablet (500 μg, t.i.d.)	3.82 ± 1.04 y	3.77 ± 1.12 y	1. CER (*p* < 0.05) 2. MDNS (*p* < 0.01) 2. MMNCV (*p* > 0.05) 3. MSNCV (*p* > 0.05) 4. PMNCV (*p* < 0.05) 5. PSNCV (*p* < 0.05) 6. TMNCV (*p* < 0.05) 7. TSNCV (*p* < 0.05)	8 w	Trial: No AE Control: No AE
Wu (2019) [76]	DPN	RCT	30(16/14) 57.60 ± 7.20 y	30(16/14) 57.03 ± 7.63 y	Taohongsiwu decoction (t.i.d.)	Epalrestat tablet (50 mg, t.i.d.)	4.3 y	4.3 y	1. CER (*p* < 0.05) 2. MSNCV (*p* < 0.05) 3. PSNCV (*p* < 0.05)	4 w	Trial: No AE Control: No AE
Yi (2019) [77]	DPN	RCT	60(31/29) 61.36 ± 4.37 y	60(29/31) 61.53 ± 4.64 y	Mongolian medicine zhenbo pill (0.2 g × 15 p, b.i.d.)	α-Lipoic acid tablet (0.3 g × 2 c, q.d.)	8.23 ± 3.21 y	8.23 ± 3.12 y	1. MDNS (*p* < 0.05) 2. MMNCV (*p* < 0.05) 3. MSNCV (*p* < 0.05) 4. PMNCV (*p* < 0.05) 5. PSNCV (*p* < 0.05)	24 w	Trial: 5 AEs/nausea (2), anorexia (3) Control: 6 AEs/ nausea (2), gastric pain (2)
Ji (2019) [78]	DPN	RCT	54(32/22) 54.47 ± 9.81 y	53(33/20) 54.81 ± 9.44 y	1. Yangyinzhuyu decoction (150 mL, b.i.d.) 2. Epalrestat tablet (50 mg, t.i.d.)	Epalrestat tablet (50 mg, t.i.d.)	10.24 ± 3.08 y	10.53 ± 2.66 y	1. CER (*p* < 0.05)	90 d	Trial: No AE Control: No AE
Liu (2019a) [79]	DPN	RCT	40 Other information NR	40 Other information NR	1. Shengjinsan combined Taohongyin (200 mL, b.i.d.) 2. Mecobalamin tablet (500 mg, t.i.d.)	Mecobalamin tablet (500 mg, t.i.d.)	NR	NR	1. MMNCV (*p* < 0.05) 2. MSNCV (*p* < 0.05) 3. TMNCV (*p* < 0.05) 4. TSNCV (*p* < 0.05)	4 w	NR
Liu (2019b) [80]	DPN	RCT	45(27/18) 58.77 ± 4.26 y	45(26/19) 59.46 ± 4.77 y	1. Huangqiguizhiwuwu decoction (400 mL, b.i.d.) 2. Epalrestat tablets (t.i.d.) 3. Mecobalamin tablet (t.i.d.)	1. Epalrestat tablets (t.i.d.) 2. Mecobalamin tablet (t.i.d.)	3.28 ± 1.45 m	3.31 ± 1.13 m	1. CER (*p* < 0.05)	8 w	NR
Chen (2021) [81]	DPN	RCT	28(15/13) 57.2 ± 8.1 y	29(16/13) 56.5 ± 7.6 y	1. Zicuijuanbi decoction (150 mL, b.i.d.) 2. Normal saline injection (250 mL, i.v.)	1. gabapentin capsule (0.3 g, t.i.d.) 2. Normal saline injection (250 mL, i.v.)	15.57 ± 3.68 y	14.59 ± 4.35 y	1. VAS (*p* < 0.05) 2. PSNCV (*p* < 0.05) 3. CER (*p* < 0.05)	10 w	NR
Hou (2021) [82]	DPN	RCT	39(24/15) 56.74 ± 11.79 y	28(18/10) 55.83 ± 10.60 y	Jiuchongdan (40 pills, t.i.d.)	Mecobalamin tablet (500 μg, t.i.d.)	15.28 ± 11.23 m	16.72 ± 10.96 m	1. CER (*p* < 0.05) 2. PSNCV (*p* < 0.05) 3. MSNCV (*p* < 0.05) 4. USNCV (*p* < 0.05)	12 w	NR
Jin (2021) [83]	DPN	RCT	51(NR) 64.36 ± 7.08 y	53(NR) 62.23 ± 7.32 y	Shenxiezhitoing capsule (3 c, t.i.d.)	α-Lipoic acid tablet (0.3 g × 2 t, q.d.)	173.48 ± 84.97 m	145.67 ± 70.68 m	1. TCSS (*p* < 0.01) 2. VAS (*p* < 0.05)	12 w	NR
Li (2021) [84]	DPN	RCT	41(22/19) 59.81 ± 5.63 y	41(23/18) 60.20 ± 5.62 y	1. Huangqiguizhiwuwu decoction (200 mL, t.i.d.) combined Mudan granule (7 g, t.i.d.) 2. Mecobalamin tablet (500 mg, t.i.d.)	1. Mecobalamin tablet (500 mg, t.i.d.)	3.15 ± 0.45 y	3.12 ± 0.43 y	1. CER (*p* < 0.05) 2. MMNCV (*p* < 0.05) 3. MSNCV (*p* < 0.05) 4. PMNCV (*p* < 0.05) 5. PSNCV (*p* < 0.05)	8 w	Trial: 5 AEs/diarrhea (1), nausea (1), constipation (2), dizziness (1) Control: 1 AE/ nausea (1)
Wang (2021a) [85]	DPN	RCT	30(16/14) 64.63 ± 4.72 y	30(17/13) 64.71 ± 4.68 y	1. Yiqiyangyintongluo decoction (200 mL, b.i.d.) 2. Epalrestat tablets (50 mg, t.i.d.)	1. Epalrestat tablets (50 mg, t.i.d.)	6.14 ± 1.24 y	6.12 ± 1.22 y	1. CER (*p* < 0.05) 2. TCSS (*p* < 0.05)	12 w	NR
Wang (2021b) [86]	DPN	RCT	50(34/16) 67.13 ± 6.29 y	50(32/18) 67.13 ± 6.29 y	1. Taohongsiwu decoction (b.i.d.) 2. Mecobalamin capsule (0.5 mg, t.i.d.)	1. Mecobalamin capsule (0.5 mg, t.i.d.)	1.57 ± 0.51 y	1.42 ± 0.83 y	1. TCSS (*p* < 0.05) 2. MMNCV (*p* < 0.05) 3. MSNCV (*p* < 0.05) 4. PMNCV (*p* < 0.05) 5. PSNCV (*p* < 0.05) 6. TMNCV (*p* < 0.05) 7. TSNCV (*p* < 0.05)	4 w	NR
Zhang (2021) [87]	DPN	RCT	74 Total 148(78/70) 59.64 ± 8.94 y	74 Total 148(78/70) 59.64 ± 8.94 y	1. Buqizhitoing decoction (b.i.d.) 2. α-Lipoic acid injection (0.6 g, q.d.) combined 0.9% Sodium chrolide injection (250 mL, q.d.)	1. α-Lipoic acid injection (0.6 g, q.d.) combined 0.9% Sodium chloride injection (250 mL, q.d.)	Total 9.33 ± 1.25 y	Total 9.33 ± 1.25 y	1. TSNCV (*p* < 0.05) 2. PSNCV (*p* < 0.05) 3. TCSS (*p* < 0.05) 4. NRS (*p* < 0.05)	8 w	NR
Nishioka (2011) [88]	CIPN	RCT	22(14/8) 67(48–77)	23(8/15) 65(52–80)	Goshajinkigan (2.5 g, t.i.d.)	No treatment	NR	NR	Incidence rate (*p*-value NR)	20 course chemotherapy	Adverse events unrelated to EAHM were reported.
Huang (2013) [89]	CIPN	RCT	30(17/13) 62.30 ± 8.29 y	31(21/10) 60.00 ± 8.88 y	Yiqiwenjingyangxuehuoxue recipe (200 mL, b.i.d.)	No treatment	NR	NR	Incidence rate (*p* < 0.05)	4 w	NR
Abe (2013) [90]	CIPN	RCT	33(NR) median 58(35–70)	27(NR) median 55(33–69)	Goshajinkigan (2.5 g, b.i.d. or t.i.d.)	Mecobalamin tablet (500 μg, t.i.d.)	NR	NR	Incidence rate (*p* < 0.01) 2. VAS (*p* < 0.01)	18 w	Adverse events unrelated to EAHM were reported.
Kono (2013) [91]	CIPN	RCT	44(23/21) median 67(40–88)	45(25/20) median 61(36–82)	Goshajinkigan (2.5 g, b.i.d. or t.i.d.)	Placebo	NR	NR	Incidence rate (*p*-value NR)	26 w	Adverse events unrelated to EAHM were reported.
Li (2013) [92]	CIPN	RCT	30(9/21) 52.1 ± 11.50 y	45(25/20) 54.4 ± 11.09	Rongjin fang decoction (200 mL, b.i.d.)	Glutathione injection (1500 mg/m^2^, q.d., i.v. drip)	9.1 ± 2.42m	8.3 ± 3.02m	Incidence rate (*p* < 0.005)	24 w	Adverse events unrelated to EAHM were reported.
Oki (2015) [93]	CIPN	RCT	89(48/41) 62.4 ± 10.6 y	93(51/42) 60.4 ± 11.5 y	Goshajinkigan (2.5 g, b.i.d. or t.i.d.)	Placebo	NR	NR	Incidence rate (*p* < 0.05)	12 course chemotherapy	Adverse events unrelated to EAHM were reported.
Xu (2017) [94]	CIPN	RCT	34(19/15) 52.4 ± 8.1 y	34(20/14) 51.8 ± 7.6 y	Modified huangqiguizhiwuwu decoction (b.i.d.)	Mecobalamin tablet (500 μg, t.i.d.)	NR	NR	Incidence rate (*p* < 0.05)	4 course chemotherapy/56d	NR
Xie (2018) [95]	CIPN	RCT	30(16/14) 57.92 ± 7.33 y	30(17/13) 58.97 ± 6.20 y	1. Yiqihuoxue decoction (500 mL, t.i.d.) 2. Duloxetine (30 mg, t.i.d.) 3. Gabapentine (600 mg, t.i.d.)	1. Duloxetine (30 mg, t.i.d.) 2. Gabapentine (600 mg, t.i.d.)	27.65 ± 9.06 d	28.16 ± 7.53 d	1. CER (*p* < 0.05) 2. MMNCV (*p* < 0.05) 3. MSNCV (*p* < 0.05)	12 w	NR
Liu (2018) [96]	CIPN	RCT	41(25/16) 62.54 ± 7.86 y	41(22/19) 61.69 ± 8.34 y	Yiqiwenyangtougluo decoction (300 mL, b.i.d.)	Amifostine injection (500 mg/m^2^, i.v. drip)	NR	NR	1. Incidence rate (*p* < 0.05) 2. MMNCV (*p* < 0.05) 3. MSNCV (*p* > 0.05) 4. SMNCV (*p* < 0.05) 5. SSNCV (*p* > 0.05)	24 w	NR
Zhang (2018) [97]	CIPN	RCT	40(24/16) 56.27 ± 9.22 y	40(23/17) 56.80 ± 9.42 y	Self-prescribed herbal medicine (q.d.)	No treatment	NR	NR	Incidence rate (*p* < 0.05)	4 course chemotherapy/4 w	NR
Liu (2020) [98]	CIPN	RCT	40(28/12) 56.2 ± 8.4 y	42(30/12) 52.8 ± 10.5 y	Bushenhuoxue herbal medicine (b.i.d.)	Dexamethasone injection (40 mg, i.v. drip)	NR	NR	Incidence rate (*p* < 0.05)	6 course chemotherapy/18 w	NR
Li (2016c) [99]	PHN	RCT	25(12/13) 58.31 ± 7.95 y	25(13/12) 58.31 ± 8.11 y	Self-prescribed Jingdutongluo decoction (t.i.d.)	Cobamamide injection (1.5 mg, q.d., i.m.)	7.52 ± 2.16 m	7.58 ± 2.38 m	1. CER (*p* < 0.05) 2. VAS (*p* < 0.05)	4 w	NR
Zhang (2012) [100]	PHN	RCT	30(16/14) median 58.32 y	30(17/13) median 59.38 y	Modified chushiweiling decoction (b.i.d.)	1. Vitamin B1 (10 mg, t.i.d.) 2. Mecobalamin tablet (0.5 mg, t.i.d.)	6.8 d	7.5 d	1. CER (*p* < 0.05) 2. VAS (*p* < 0.05)	4 w	Trial: No AE Control: No AE
Zhao (2018) [101]	PHN	RCT	47(29/18) 48.2 ± 9.4 y	46(24/22) 48.5 ± 9.6 y	Shuganzhuyuzhentong decoction (300 mL, b.i.d.)	1. Calamine lotion 2. Diclofenac sodium emulsion 3. Vitamin B 4. Mecobalamin 5. Oxycodoen hydrochloride sustained release tablet (10 mg, b.i.d.)	52.4 ± 10.9 d	48.5 ± 9.6 d	VAS improvement rate (*p* < 0.05)	4 w	Trial: 10 AEs constipation (3) nausea and vomiting (2) dizziness (2) xerostomia (2) Control: 14 AEs constipation (9) nausea and vomiting (1) dizziness (1) xerostomia(3)
Gong (2021) [102]	Occipital neuralgia	RCT	30 (16/14) 42.6 ± 6.1 y	30 (17/13) 43.2 ± 6.4 y	1. Modified chuanxiongchadio san 2. Gabapentin capsule (0.3 g, t.i.d.)	1. Gabapentin capsule (0.3 g, t.i.d.)	4.2 ± 1.1 d	4.6 ± 1.3 d	1. CER (*p* < 0.05) 2. VAS (*p* < 0.05)	2 w	NR
Huang (2020) [103]	Trigeminal neuralgia	RCT	30 (15/15) 58.50 ± 10.72 y	30 (9/21) 60.07 ± 13.57 y	1. Xiongzhiyufeng decoction (b.i.d.) 2. Carbamazepine (0.1 g, b.i.d.)	1. Carbamazepine (0.1 g, b.i.d.)	2.95 ± 3.19 y	2.12 ± 2.46 y	1. CER (*p* < 0.05) 2. VAS (*p* < 0.05)	20 d	NR
Song (2020) [104]	Supraorbital neuralgia	RCT	45(NR) 52.2 ± 3.5 y	42(NR) 50.1 ± 4.2 y	Yangxueshugan decoction (b.i.d.)	1. Mecobalamin tablet (500 μg, t.i.d.) 2. Citicoline sodium (q.d.)	NR	NR	1. CER (*p* < 0.05)	2 w	NR

AE: Adverse event; b.i.d: Bis in die; c: Capsules; CER: Clinical effective rate; CIPN: Chemotherapy-induced peripheral neuropathy; d: Days; DPN: Diabetic peripheral neuropathy; g: Gram; i.m.: Intramuscular; i.v.: Intravenous; m: Months; MDNS: Michigan diabetic neuropathy scale; mg: Milligram; mL: Milliliter; MMNCV: Median motor nerve conduction velocity; MSNCV: Median sensory nerve conduction velocity; NR: Not reported; p.o: Per os; PHN: Postherpetic neuralgia; PMNCV: Peroneal motor nerve conduction velocity; PSNCV: Peroneal sensory nerve conduction velocity; q.d.: Quaque die; RCT: Randomized controlled trial; SD: Standard deviation; SMNCV: Sural motor nerve conduction velocity; SSNCV: Sural sensory nerve conduction velocity; t: Tablet; t.i.d: Ter in die; TCSS: Toronto clinical scoring scale; TMNCV: Tibial motor nerve conduction velocity; TSNCV: Tibial sensory nerve conduction velocity; UMNCV: Ulnar motor nerve conduction velocity; USNCV: Ulnar motor nerve conduction velocity; y: Years; µg: Microgram.

**Table 2:** Methodological quality of the included studies according to the risk of bias 2.0.

Author (Year) [Reference]	D1	D2	D3	D4	D5	Overall
Jin (2004) [38]	Sc	H	H	L	Sc	H
Sun (2008) [39]	Sc	H	H	Sc	Sc	H
Shen (2009) [40]	L	H	H	L	Sc	H
Lin (2010) [41]	Sc	H	H	L	Sc	H
Wang (2010) [42]	L	H	H	L	Sc	H
Yan (2010) [43]	Sc	H	H	Sc	Sc	H
Wu (2011) [44]	Sc	H	H	L	Sc	H
Gao (2012) [45]	Sc	H	H	L	Sc	H
Gong (2013) [46]	Sc	H	H	L	Sc	H
Han (2013) [47]	L	H	H	Sc	Sc	H
Zhang (2013a) [48]	L	H	H	L	Sc	H
Zhang (2013b) [49]	Sc	H	H	L	Sc	H
Guo (2014) [50]	Sc	H	H	Sc	Sc	H
Yang (2014a) [51]	Sc	H	H	Sc	Sc	H
Yang (2014b) [52]	L	H	H	Sc	Sc	H
Qi (2015) [53]	Sc	H	H	L	Sc	H
Wang (2015) [54]	Sc	H	H	Sc	Sc	H
Xue (2015) [55]	L	H	H	L	Sc	H
Ding (2016) [56]	Sc	H	H	L	Sc	H
Guo (2016) [57]	L	H	H	L	Sc	H
Han (2016) [58]	Sc	H	H	L	Sc	H
Lan (2016) [59]	H	H	H	L	Sc	H
Mo (2016) [60]	L	H	H	Sc	Sc	H
Wang (2016) [61]	L	H	H	Sc	Sc	H
Li (2016a) [62]	L	H	H	L	Sc	H
Zhang (2016a) [63]	L	H	H	L	Sc	H
Li (2016b) [64]	Sc	H	H	L	Sc	H
Zhang (2016b) [65]	L	H	H	L	Sc	H
Chen (2017) [66]	L	H	H	L	Sc	H
Shi (2017) [67]	Sc	H	H	L	Sc	H
Wang (2017) [68]	L	H	H	L	Sc	H
Chen (2018) [69]	L	H	H	Sc	Sc	H
Dai (2018) [70]	H	H	H	L	Sc	H
Hu (2018) [71]	Sc	H	H	L	Sc	H
Huang (2018) [72]	L	H	H	L	Sc	H
She (2018) [73]	L	H	H	L	Sc	H
Xin (2018) [74]	H	H	H	Sc	Sc	H
Gao (2019) [75]	Sc	H	H	L	Sc	H
Wu (2019) [76]	L	H	H	L	Sc	H
Yi (2019) [77]	L	H	H	L	Sc	H
Ji (2019) [78]	L	H	H	Sc	Sc	H
Liu (2019a) [79]	Sc	H	H	L	Sc	H
Liu (2019b) [80]	Sc	H	H	Sc	Sc	H
Chen (2021) [81]	Sc	H	Sc	L	Sc	H
Hou (2021) [82]	Sc	H	H	L	Sc	H
Jin (2021) [83]	L	H	Sc	L	Sc	H
Li (2021) [84]	L	H	H	L	Sc	H
Wang (2021a) [85]	L	H	H	L	Sc	H
Wang (2021b) [86]	L	H	H	L	Sc	H
Zhang (2021) [87]	H	H	H	L	Sc	H
Nishioka (2011) [88]	L	L	L	L	L	L
Huang (2013) [89]	L	H	Sc	L	Sc	H
Abe (2013) [90]	L	L	L	L	L	L
Kono (2013) [91]	L	L	L	L	L	L
Li (2013) [92]	L	H	H	L	Sc	H
Oki (2015) [93]	L	L	L	L	L	L
Xu (2017) [94]	Sc	H	H	L	Sc	H
Xie (2018) [95]	L	H	H	L	Sc	H
Liu (2018) [96]	L	H	H	L	Sc	H
Zhang (2018) [97]	Sc	H	H	L	Sc	H
Liu (2020) [98]	Sc	H	H	L	Sc	H
Li (2016c) [99]	L	H	H	L	Sc	H
Zhang (2012) [100]	Sc	H	H	L	Sc	H
Zhao (2018) [101]	L	H	H	Sc	Sc	H
Gong (2021) [102]	Sc	H	H	L	Sc	H
Huang (2020) [103]	Sc	H	H	L	Sc	H
Song (2020) [104]	H	H	H	Sc	Sc	H

D1–D5: 5 Domain criteria. D1: Bias arising from the randomization process; D2: Bias due to deviations from the intended interventions; D3: Bias due to the missing outcome data; D4: Bias in the measurement of the outcome; and D5: Bias in the selection of the reported results. H: High risk of bias; L: Low risk of bias; Sc: Some concerns.

**Table 3:** Subgroup analysis for patient type and nerve conduction velocity outcome.

Intervention and Comparator	Outcomes	Subgroup Analysis		Number of Participants (Studies)	Mean Difference (95% CI)	Heterogeneity	
						I^2^, %	*p*
EAHM in combination with the other treatment vs. active control	MSNCV	Main analysis		1333(14)	2.73 (1.80 to 3.66)	86%	*p* < 0.01
		Patient types	DPN	1191(12)	2.80 (1.83 to 3.78)	85%	*p* < 0.01
			CIPN	142(2)	2.27 (−1.93 to 6.48)	95%	*p* < 0.01
		Duration of treatment	≤4 weeks	635(6)	3.01 (1.20 to 4.82)	93%	*p* < 0.01
			>4 weeks, ≤11 weeks	302(4)	2.31 (1.05 to 3.56)	73%	*p* = 0.01
			>11 weeks	396(4)	2.81 (0.84 to 4.78)	74%	*p* < 0.01
	PSNCV	Main analysis		1329(14)	2.59 (1.65 to 3.53)	88%	*p* < 0.01
		Patient types	DPN	1247(13)	2.79 (1.81 to 3.76)	88%	*p* < 0.01
			CIPN	82(1)	0.35 (−0.66 to 1.36)	-	-
		Duration of treatment	≤4 weeks	364(4)	3.02 (2.29 to 3.76)	0%	*p* = 0.57
			>4 weeks, ≤11 weeks	235(3)	2.64 (0.10 to 5.19)	95%	*p* < 0.01
			>11 weeks	630(7)	2.49 (1.09 to 3.89)	89%	*p* < 0.01
	TSNCV	Main analysis		1214(11)	3.59 (2.79 to 4.39)	81%	*p* < 0.01
		Patient types	Only DPN	-	-	-	-
		Duration of treatment	≤4 weeks	440(4)	4.60 (2.38 to 6.82)	85%	*p* < 0.01
			>4 weeks, ≤11 weeks	166(2)	2.13 (1.62 to 2.63)	0%	*p* = 0.73
			>11 weeks	608(5)	3.72 (3.11 to 4.32)	33%	*p* = 0.20
	MMNCV	Main analysis		980(10)	3.31 (1.96 to 4.65)	88%	*p* < 0.01
		Patient types	DPN	828(8)	3.49 (2.10 to 4.89)	84%	*p* < 0.01
			CIPN	142(2)	2.64 (−2.26 to 7.54)	97%	*p* < 0.01
		Duration of treatment	>11 weeks	528(5)	4.21 (2.18 to 6.24)	92%	*p* < 0.01
			>4 weeks, ≤11 weeks	142(2)	1.88 (0.78 to 2.98)	0%	*p* = 0.61
			≤4 weeks	300(3)	2.68 (1.51 to 3.85)	9%	*p* = 0.33
	PMNCV	Main analysis		1234(13)	2.98 (2.12 to 3.85)	81%	*p* < 0.01
		Patient types	DPN	1152(12)	3.22 (2.43 to 4.01)	73%	*p* < 0.01
			CIPN	82(1)	0.35 (−0.66 to 1.36)	-	-
		Duration of treatment	≤4 weeks	460(5)	4.42 (3.51 to 5.33)	29%	*p* = 0.23
			>4 weeks, ≤11 weeks	450(4)	1.91 (0.79 to 3.02)	75%	*p* < 0.01
			>11 weeks	324(4)	2.48 (1.29 to 3.68)	63%	*p* = 0.04
EAHM monotherapy vs. active control	MSNCV	Main analysis		681(7)	2.74 (1.38 to 4.10)	89%	*p* < 0.01
		Patient types	Only DPN	-	-	-	-
		Duration of treatment	≤4 weeks	144(2)	2.46 (−0.47 to 5.39)	96%	*p* < 0.01
			>4 weeks, ≤11 weeks	303(2)	3.72 (−0.15 to 7.59)	95%	*p* < 0.01
			>11 weeks	234(3)	2.23 (0.75 to 3.71)	25%	*p* = 0.26
	PSNCV	Main analysis		883(10)	2.76 (1.67 to 3.85)	85%	
		Patient types	Only DPN	-	-	-	-
		Duration of treatment	≤4 weeks	224(3)	3.46 (2.04 to 4.88)	77%	*p* = 0.01
			>4 weeks, ≤11 weeks	425(4)	2.23 (0.09 to 4.38)	89%	*p* < 0.01
			>11 weeks	234(3)	2.73 (0.45 to 5.02)	73%	*p* = 0.02
	PMNCV	Main analysis		967(9)	2.47 (1.40 to 3.53)	75%	*p* < 0.01
		Patient types	Only DPN	-	-	-	-
		Duration of treatment	≤4 weeks	80(1)	3.61 (1.43 to 5.79)	-	-
			>4 weeks, ≤11 weeks	725(6)	2.37 (0.91 to 3.83)	83%	*p* < 0.01
			>11 weeks	162(2)	2.40 (0.85 to 3.96)	35%	*p* = 0.21

CIPN: Chemotherapy-induced peripheral neuropathy; DPN: Diabetic peripheral neuropathy; EAHM: East Asian herbal medicine; MSNCV: Median sensory nerve conduction velocity; PSNCV: Peroneal sensory nerve conduction velocity; TSNCV: Tibial sensory nerve conduction velocity; MMNCV: Median motor nerve conduction velocity; PMNCV: Peroneal motor nerve conduction velocity.

**Table 4:** The top 10 frequent herbs prescribed for peripheral neuropathy.

EAHM (Latin Name)	Frequency of Utilization	Relative Frequency (%)	Cumulative Percentiles (%)
Astragali Radix	42	6.27	6.27
Angelicae Gigantis Radix	33	4.93	11.20
Paeoniae Radix	33	4.93	16.13
Cnidii Rhizoma	29	4.33	20.46
Cinnamomi Ramulus	28	4.18	24.64
Spatholobi Caulis	24	3.58	28.22
Achyranthis Radix	20	2.99	31.21
Glycyrrhyziae Radix et Rhizoma	20	2.99	34.20
Salviae Miltiorrhizae Radix	20	2.99	37.19
Carthami Flos	18	2.69	39.88

EAHM: East Asian herbal medicine.

**Table 5:** Apriori algorithm-based association rules for EAHM prescribed for peripheral neuropathy.

No.	Associations Rules	Support	Confidence	Lift
1	{Glycyrrhizae Radix et Rhizoma} => {Astragali Radix}	0.239	0.800	1.276
2	{Spatholobi Caulis} => {Astragali Radix}	0.313	0.875	1.396
3	{Cinnamomi Ramulus} => {Astragali Radix}	0.373	0.893	1.424
4	{Cinnamomi Ramulus, Spatholobi Caulis} => {Astragali Radix}	0.254	0.944	1.507
5	{Astragali Radix, Spatholobi Caulis} => {Cinnamomi Ramulus}	0.254	0.810	1.937
6	{Angelicae Gigantis Radix, Cinnamomi Ramulus} => {Paeoniae Radix}	0.224	0.882	1.791
7	{Cinnamomi Ramulus, Paeoniae Radix} => {Astragali Radix}	0.284	0.864	1.378
8	{Angelicae Gigantis Radix, Cinnamomi Ramulus} => {Astragali Radix}	0.209	0.824	1.314
9	{Cnidii Rhizoma, Paeoniae Radix} => {Angelicae Gigantis Radix}	0.239	0.842	1.710

**Table 6:** Summary of findings for the studies in this meta-analysis.

Intervention and Comparator Intervention	Outcomes	Number of Participants (Studies)	Anticipated Absolute of Relative Effects (95% CI)	Quality of the Evidence (GRADE)
EAHM combination of WM compared to WM for peripheral neuropathy	SNCV	4454 (21 RCTs)	MD 3.06 higher (2.56 higher to 3.56 higher)	⨁⨁⨁◯ MODERATE
	MNCV	2860 (16 RCTs)	MD 3.23 higher (2.58 higher to 3.88 higher)	⨁⨁⨁◯ MODERATE
	Response rate	1997 (25 RCTs)	RR 1.20 (1.15 to 1.25)	⨁⨁⨁◯ MODERATE
	Incidence rate	232 (3 RCTs)	OR 0.12 (0.03 to 0.59)	⨁⨁◯◯ LOW
	Pain intensity	332 (4 RCTs)	SMD 1.21 SD lower (1.29 lower to 0.83 lower)	⨁⨁⨁◯ MODERATE
	TCSS	470 (4 RCTs)	MD 1.83 lower (2.11 lower to 1.55 lower)	⨁⨁◯◯ LOW
	MDNS	122 (2 RCTs)	MD 2.21 lower (2.94 lower to 1.47 lower)	⨁⨁◯◯ LOW
EAHM monotherapy compared WM for peripheral neuropathy	SNCV	2159 (10 RCTs)	MD 2.68 higher (2.02 higher to 3.35 higher)	⨁⨁⨁◯ MODERATE
	MNCV	1788 (9 RCTs)	MD 2.38 higher (1.43 higher to 3.32 higher)	⨁⨁⨁◯ MODERATE
	Response rate	1651 (21 RCTs)	RR 1.30 (1.20 to 1.29)	⨁⨁◯◯ LOW
	Incidence rate	249 (4 RCTs)	OR 0.17 (0.07 to 0.38)	⨁⨁◯◯ LOW
	Pain intensity	294 (4 RCTs)	SMD 0.94 SD lower (1.18 lower to 0.69 lower)	⨁⨁⨁◯ MODERATE
	TCSS	187 (3 RCTs)	MD 1.04 lower (1.75 lower to 0.34 lower)	⨁⨁◯◯ LOW
	MDNS	207 (2 RCTs)	MD 2.95 lower (4.2 lower to 1.7 lower)	⨁⨁◯◯ LOW

EAHM: East Asian herbal medicine; MD: Mean difference; MDNS: Michigan diabetic neuropathy score; MNCV: Motor nerve conduction velocity; RCT: Randomized clinical trial; SNCV: Sensory nerve conduction velocity; SMD: Standardized mean difference; TCSS: Toronto clinical scoring system; OR: Odds ratio; RR: Risk ratio; CI: Confidence interval. Working group grades of Evidence. High quality: Further research is very unlikely to change our confidence in the estimate of effect. Moderate quality: Further research is likely to have an important impact on our confidence in the estimate of effect and may change the estimate. Low quality: Further research is very likely to have an important impact on our confidence in the estimate of effect and is likely to change the estimate. Very low quality: Very uncertain about the estimate.

## Data Availability

Data sharing not applicable.

## Supplementary Materials

The following are available online at https://www.mdpi.com/article/10.3390/ph14111202/s1. Table S1: PRISMA_2020_checklist; Table S2: Search strategy; Table S3: The Ingredients of EAHM used in clinical trials included in this study.
