# Supplementary material for: Oral Administration of East Asian Herbal Medicine for Peripheral Neuropathy: A Systematic Review and Meta-Analysis with Association Rule Analysis to Identify Core Herb Combinations
Source: Pharmaceuticals (Basel). 2021 Nov 22;14(11):1202. doi: 10.3390/ph14111202 (PMC8622183; doi:10.3390/ph14111202)
Supplement: Supplementary file 1 [file pharmaceuticals-14-01202-s001.zip › Table S2. Search strategy.pdf]

Table S2. Search strategies

MEDLINE

Searching strategy	
#1	mononeuropathy[MeSH] OR nerve compression syndromes[MeSH] OR neuralgia[MeSH] OR polyneuropathies [MeSH]
#2	“neuropathy”[Title/abstract] OR “peripheral neuropathy”[Title/abstract] OR “neuropathic pain”[Title/abstract] OR “neuralgia”[Title/abstract]
#3	“Plants, Medicinal”[MeSH] OR “Drugs, Chinese Herbal”[MeSH] OR “Medicine, Chinese Traditional”[MeSH] OR “Medicine, Kampo”[MeSH] OR “Medicine, Korean Traditional”[MeSH] OR “Herbal Medicine”[MeSH] OR “Prescription Drugs”[MeSH] OR “traditional Korean medicine”[Title/abstract] OR “traditional Chinese medicine”[Title/abstract] OR “traditional oriental medicine”[Title/abstract] OR “Kampo medicine”[Title/abstract] OR herb*[Title/abstract] OR decoction*[Title/abstract] OR botanic*[Title/abstract]
#4	#1 AND #2 AND #3

EMBASE

Searching strategy	
#1	('neuralgia'/exp OR neuralgia OR 'mononeuropathy'/exp OR mononeuropathy OR 'nerve'/exp OR nerve) AND ('compression'/exp OR compression) AND syndromes OR 'polyneuropathies'/exp OR polyneuropathies
#2	‘peripheral neuropathy’/exp OR ‘neuropathy’ OR ‘neuropathic pain’ OR ‘neuralgia’
#3	‘medicinal plant’/exp OR ‘medicinal plant’ OR ‘herbaceous agent’/exp OR ‘herbaceous agent’ OR ‘chinese medicine’/exp OR ‘chinese medicine’ OR ‘kampo medicine’/exp OR ‘kampo medicine’ OR ‘kampo medicine (drug)’/exp OR ‘kampo medicine (drug)’ OR ‘korean medicine’/exp OR ‘korean medicine’ OR ‘herbal medicine’/exp OR ‘herbal medicine’ OR ‘prescription drug’/exp OR ‘prescription drug’ OR ‘oriental medicine’/exp OR ‘oriental medicine’ OR ‘alternative medicine’/exp OR ‘alternative medicine’ OR ‘complementary medicine’ OR ‘herb’/exp OR ‘herb’ OR ‘decoction’ OR ‘botanic’
#4	#1 AND #2 AND #3

CENTRAL

Searching strategy	
#1	MeSH descriptor: [Mononeuropathies] explode all trees
#2	(Mononeuropathies):ti,ab,kw
#3	MeSH descriptor: [Neuralgia] explode all trees

#4	(“neuropathy” OR “peripheral neuropathy” OR “neuropathic pain” OR “neuralgia”):ti,ab,kw
#5	MeSH descriptor: [Plants, Medicinal] explode all trees
#6	MeSH descriptor: [Drugs, Chinese Herbal] explode all trees
#7	MeSH descriptor: [Medicine, Chinese Traditional] explode all trees
#8	MeSH descriptor: [Medicine, Kampo] explode all trees
#9	MeSH descriptor: [Medicine, Korean Traditional] explode all trees
#10	MeSH descriptor: [Herbal Medicine] explode all trees
#11	MeSH descriptor: [Prescription Drugs] explode all trees
#12	(“traditional Korean medicine” OR “traditional Chinese medicine” OR “Traditional oriental medicine” OR “Kampo medicine” OR herb* OR decoction* OR botanic*):ti,ab,kw
#13	(#1 OR #2) AND (#3 OR #4) AND (#5 OR #6 OR #7 OR #8 OR #9 OR #10 OR #11 OR #12) in Trials
