# Supplementary material for: Oral Administration of East Asian Herbal Medicine for Peripheral Neuropathy: A Systematic Review and Meta-Analysis with Association Rule Analysis to Identify Core Herb Combinations
Source: Pharmaceuticals (Basel). 2021 Nov 22;14(11):1202. doi: 10.3390/ph14111202 (PMC8622183; doi:10.3390/ph14111202)
Supplement: Supplementary file 1 [file pharmaceuticals-14-01202-s001.zip › Table S3. The Ingredients of EAHM used in clinical trials included in this study.pdf]

Table S3. The Ingredients of EAHM used in clinical trials included in this study

| References | Condition | Ingredients of EAHM (Latin name) |
|---|---|---|
| Jin 2004 | DPN | Astragali Radix, Scrophulariae Radix, Salviae Miltiorrhizae Radix, Draconis Sanguis, Brassicae Semen, Arisaematis Rhizoma |
| Sun 2008 | DPN | Rehmanniae Radix Preparata, Moutan Radicis Cortex, Poria Sclerotium, Alismatis Rhizoma, Dioscoreae Rhizoma, Corni Fructus, Persicae Semen, Carthami Flos, Angelicae Gigantis Radix, Cnidii Rhizoma, Achyranthis Radix, Salviae Miltiorrhizae Radix, Polygoni Cuspidati Rhizoma et Radix, Spatholobi Caulis, Lumbricus |
| Shen 2009 | DPN | Acori Graminei Rhizoma, Puerariae Radix, Achyranthis Radix, Coptidis Rhizoma, Salviae Miltiorrhizae Radix, Dipsaci Radix, Leonuri Herba |
| Hong 2010 | DPN | Astragali Radix, Cinnamomi Ramulus, Zingiberis Rhizoma Recens, Zizyphi Fructus, Glycyrrhizae Radix et Rhizoma |
| Lin 2010 | DPN | Ginseng Radix, Eupolyphaga, Hirudo, Scorpio, Cicadidae Periostracum, Scolopendra, Dalbergiae Odoriferae Lignum |
| Wang 2010 | DPN | Astragali Radix, Paeoniae Radix, Cinnamomi Ramulus, Zingiberis Rhizoma Recens, Zizyphi Fructus |
| Yan 2010 | DPN | Rehmanniae Radix Recens, Coptidis Rhizoma, Puerariae Radix, Euonymi Ramuli Suberalatum, Campsitis Flos |

| | | |
|---|---|---|
| Wu 2011 | DPN | Astragali Radix, Puerariae Radix, Dioscoreae Rhizoma, Trichosanthis Radix, Scrophulariae Radix, Atractylodis Rhizoma Alba, Poria Sclerotium, Cnidii Rhizoma, Moutan Radicis Cortex, Angelicae Gigantis Radix |
| Gao 2012 | DPN | Aconiti Lateralis Radix Preparata, Paeoniae Radix, Poria Sclerotium, Atractylodis Rhizoma Alba, Ginseng Radix, Cnidii Rhizoma, Scorpio |
| Han 2013 | DPN | Astragali Radix, Cinnamomi Ramulus, Paeoniae Radix, Spatholobi Caulis, Glycyrrhizae Radix et Rhizoma |
| Gong 2013 | DPN | Aconiti Lateralis Radix Preparata, Paeoniae Radix, Poria Sclerotium, Atractylodis Rhizoma Alba, Cnidii Rhizoma, Scorpio |
| Zhang 2013a | DPN | Chaenomelis Fructus, Moutan Radicis Cortex, Coptidis Rhizoma, Rehmanniae Radix Preparata, Spatholobi Caulis |
| Zhang 2013b | DPN | Astragali Radix, Salviae Miltiorrhizae Radix, Polygonati Rhizoma, Atractylodis Rhizoma, Cnidii Rhizoma, Mori Ramulus, Piperis Kadsurae Caulis, Mori Radicis Cortex |
| Guo 2014 | DPN | Astragali Radix, Cinnamomi Ramulus, Paeoniae Radix, Paeoniae Radix, Salviae Miltiorrhizae Radix, Zizyphi Fructus, Zingiberis Rhizoma Recens, Spatholobi Caulis, Curcumae Longae Rhizoma |
| Yang 2014a | DPN | Panacis Quinquefolii Radix, Astragali Radix, Poria Sclerotium, Angelicae Gigantis |

| | | |
|---|---|---|
| | | Radix, Rehmanniae Radix Preparata, Corni Fructus, Bupleuri Radix, Hirudo, Cnidii Rhizoma, Spatholobi Caulis, Sappan Lignum, Cinnamomi Ramulus, Paeoniae Radix, Lonicerae Flos, Cyathulae Radix |
| Yang 2014b | DPN | Astragali Radix, Angelicae Gigantis Radix, Cinnamomi Ramulus, Spatholobi Caulis, Paeoniae Radix, Melandrii Herba, Zingiberis Rhizoma Recens, Zizyphi Fructus |
| Qi 2015 | DPN | Astragali Radix, Corydalis Tuber, Notoginseng Radix et Rhizoma, Paeoniae Radix, Salviae Miltiorrhizae Radix, Cnidii Rhizoma, Carthami Flos, Sappan Lignum, Spatholobi Caulis |
| Wang 2015 | DPN | Ginseng Radix, Liriopis seu Ophiopogonis Tuber, Schisandrae Fructus, Astragali Radix, Cnidii Rhizoma, Salviae Miltiorrhizae Radix, Crataegi Fructus, Hirudo |
| Xue 2015 | DPN | Piperis Kadsurae Caulis, Sinomeni Caulis et Rhizoma, Trachelospermi Caulis, Lonicerae Folium et Caulis, Uncariae Ramulus cum Uncus, Clematidis Radix, Zaocys, Spatholobi Caulis, Hirudo, Persicae Semen, Carthami Flos, Cinnamomi Ramulus, Astragali Radix |
| Ding 2016 | DPN | Astragali Radix, Citri Unshius Pericarpium, Spatholobi Caulis, Angelicae Gigantis Radix, Cnidii Rhizoma, Achyranthis Radix, Paeoniae Radix, Persicae Semen, Carthami Flos, Lumbricus, Scorpio |
| Guo 2016 | DPN | Astragali Radix, Spatholobi Caulis, Chaenomelis Fructus, Coicis Semen, |

| | | |
|---|---|---|
| | | Atractylodis Rhizoma, Tetrapanacis Medulla, Benincasae Semen, Mori Ramulus, Indigo Pulverata Levis, Tokoro Rhizoma, Achyranthis Radix, Phellodendri Cortex, Hirudo |
| Han 2016 | DPN | Angelicae Gigantis Radix, Rehmanniae Radix Recens, Cnidii Rhizoma, Paeoniae Radix, Paeoniae Radix, Salviae Miltiorrhizae Radix, Leonuri Herba, Aucklandiae Radix, Puerariae Radix |
| Lan 2016 | DPN | Astragali Radix, Spatholobi Caulis, Siegesbeckiae Herba, Clematidis Radix, Achyranthis Radix, Scorpio, Cinnamomi Ramulus |
| Mo 2016 | DPN | Lonicerae Flos, Astragali Radix, Scrophulariae Radix, Schisandrae Fructus, Puerariae Radix, Polygonati Odorati Rhizoma, Salviae Miltiorrhizae Radix, Litchi Semen, Glycyrrhizae Radix et Rhizoma |
| Wang 2016 | DPN | Astragali Radix, Cinnamomi Ramulus, Paeoniae Radix, Paeoniae Radix, Corydalis Tuber, Angelicae Gigantis Radix, Salviae Miltiorrhizae Radix, Spatholobi Caulis, Lumbricus, Clematidis Radix, Lycopodii Herba, Achyranthis Radix, Liriopis seu Ophiopogonis Tuber |
| Li 2016a | DPN | Aconiti Lateralis Radix Preparata, Eucommiae Cortex, Achyranthis Radix, Spatholobi Caulis, Astragali Radix, Angelicae Gigantis Radix, Codonopsis Pilosulae Radix, Brassicae Semen, Angelicae Dahuricae Radix, Lycopodii Herba, Myrrha, |

| | | |
|---|---|---|
| | | Cinnamomi Ramulus, Scolopendra, Corydalis Tuber, Cnidii Rhizoma, Lumbricus |
| Zhang 2016a | DPN | Astragali Radix, Paeoniae Radix, Saposhnikoviae Radix, Angelicae Gigantis Radix, Cinnamomi Ramulus, Tetrapanacis Medulla, Asiasari Radix et Rhizoma, Glycyrrhizae Radix et Rhizoma, Cnidii Rhizoma, Speranskiae Tuberculatae Herba, Lycopodii Herba |
| Li 2016b | DPN | Astragali Radix, Notoginseng Radix et Rhizoma, Hirudo, Clematidis Radix, Achyranthis Radix |
| Zhang 2016b | DPN | Astragali Radix, Puerariae Radix, Rehmanniae Radix, Lycii Fructus, Cassiae Semen, Leonuri Semen, Typhae Pollen, Hirudo |
| Chen 2017 | DPN | Cinnamomi Ramulus, Paeoniae Radix, Zingiberis Rhizoma Recens, Astragali Radix, Angelicae Gigantis Radix, Glycyrrhizae Radix et Rhizoma, Asiasari Radix et Rhizoma, Aconiti Lateralis Radix Preparata, Zingiberis Rhizoma, Tetrapanacis Medulla |
| Shi 2017 | DPN | Salviae Miltiorrhizae Radix, Bomeolum, Notoginseng Radix et Rhizoma |
| Wang 2017 | DPN | Cinnamomi Ramulus, Asiasari Radix et Rhizoma, Angelicae Gigantis Radix, Glycyrrhizae Radix et Rhizoma, Paeoniae Radix, Zizyphi Fructus, Tetrapanacis Medulla |
| Chen 2018 | | |

| | | |
|---|---|---|
| | DPN | Angelicae Gigantis Radix, Paeoniae Radix, Asiasari Radix et Rhizoma, Cinnamomi Ramulus, Tetrapanacis Medulla, Zizyphi Fructus, Glycyrrhizae Radix et Rhizoma |
| Dai 2018 | DPN | Paeoniae Radix, Astragali Radix, Rehmanniae Radix Preparata, Achyranthis Radix, Gypsum Fibrosum, Liriopis seu Ophiopogonis Tuber, Anemarrhenae Rhizoma, Angelicae Gigantis Radix, Cinnamomi Ramulus, Carthami Flos, Persicae Semen, Zingiberis Rhizoma Recens, Zizyphi Fructus, Scorpio, Lumbricus, Scolopendra |
| Hu 2018 | DPN | Astragali Radix, Cinnamomi Ramulus, Paeoniae Radix, Zingiberis Rhizoma Recens, Carthami Flos, Angelicae Gigantis Radix, Persicae Semen, Rehmanniae Radix Recens, Codonopsis Pilosulae Radix, Glycyrrhizae Radix et Rhizoma, Cnidii Rhizoma, Spatholobi Caulis |
| Huang 2018 | DPN | Myrrha, Achyranthis Radix, Aconiti Lateralis Radix Preparata, Astragali Radix, Mori Ramulus, Persicae Semen, Paeoniae Radix, Carthami Flos, Ephedrae Herba, Codonopsis Pilosulae Radix, Notoginseng Radix et Rhizoma, Olibanum, Eupolyphaga, Angelicae Gigantis Radix, Asiasari Radix et Rhizoma |
| She 2018 | DPN | Astragali Radix, Cinnamomi Ramulus, Paeoniae Radix, Zingiberis Rhizoma Recens, Zizyphi Fructus |
| Xin 2018 | DPN | Tuber Aconiti, Moschus, Acori Graminei Rhizoma, Aucklandiae Radix, Terminaliae Fructus, Magenetitum, Margarita, Margarita, Glycyrrhizae Radix et Rhizoma, |

| | | |
|---|---|---|
| | | Syzygii Flos, Myristicae Semen, Aquilariae Lignum, Limonitum |
| Gao 2019 | DPN | Scorpio, Euonymi Ramuli Suberalatum, Salviae Miltiorrhizae Radix, Cibotii Rhizoma, Dipsaci Radix, Astragali Radix, Liriopis seu Ophiopogonis Tuber, Corni Fructus, Ginseng Radix, Schisandrae Fructus |
| Wu 2019 | DPN | Angelicae Gigantis Radix, Paeoniae Radix, Cnidii Rhizoma, Rehmanniae Radix Preparata, Persicae Semen, Carthami Flos |
| Yi 2019 | DPN | Moschus, Dalbergiae Odoriferae Lignum, Inula racemosa, Aucklandiae Radix, Carthami Flos, Lagotis brachystachya Maxim, Piperis Longi Fructus, Syzygii Flos, Alpiniae Officinari Rhizoma, Fruttus cymini, Cinnamomi Cortex, Myristicae Semen, Margarita, Olibanum, Bubali Cornu, Bovis Calculus Artifactus, Moschus |
| Ji 2019 | DPN | Codonopsis Pilosulae Radix, Liriopis seu Ophiopogonis Tuber, Schisandrae Fructus, Astragali Radix, Salviae Miltiorrhizae Radix, Amomi Fructus, Dalbergiae Odoriferae Lignum, Asiasari Radix et Rhizoma, Angelicae Gigantis Radix, Cnidii Rhizoma, Paeoniae Radix, Carthami Flos, Achyranthis Radix, Cinnamomi Ramulus, Notoginseng Radix et Rhizoma, Lumbricus, Glycyrrhizae Radix et Rhizoma |
| Liu 2019a | DPN | Batryticatus Bombyx, Cicadidae Periostracum, Rhei Radix et Rhizoma, Curcumae Longae Rhizoma, Persicae Semen, Carthami Flos, Cnidii Rhizoma, Angelicae Gigantis Radix, Clematidis Radix |

| | | |
|---|---|---|
| Liu 2019b | DPN | Astragali Radix, Spatholobi Caulis, Zingiberis Rhizoma Recens, Atractylodis Rhizoma Alba, Cinnamomi Ramulus, Lumbricus, Codonopsis Pilosulae Radix, Paeoniae Radix, Zizyphi Fructus, Glycyrrhizae Radix et Rhizoma |
| Jiang 2020 | DPN | Spatholobi Caulis, Angelicae Gigantis Radix, Lumbricus, Rehmanniae Radix Recens, Cnidii Rhizoma, Liquidambaris Fructus, Paeoniae Radix, Persicae Semen, Carthami Flos |
| Chen 2021 | DPN | Astragali Radix, Cinnamomi Ramulus, Rehmanniae Radix Recens, Dioscoreae Rhizoma, Paeoniae Radix, Corni Fructus, Euonymi Ramuli Suberalatum, Cnidii Rhizoma, Spatholobi Caulis, Lumbricus, Corydalis Tuber, Chaenomelis Fructus, Achyranthis Radix |
| Hou 2021 | DPN | Astragali Radix, Puerariae Radix, Spatholobi Caulis, Curcumae Radi, Lumbricus, Hirudo, Cinnamomi Ramulus, Poria Sclerotium, Rehmanniae Radix Recens |
| Jin 2021 | DPN | Notoginseng Radix et Rhizoma, Scorpio, Eupolyphaga, Scolopendra |
| Li 2021 | DPN | Astragali Radix, Cinnamomi Ramulus, Angelicae Gigantis Radix, Paeoniae Radix, Cnidii Rhizoma, Salviae Miltiorrhizae Radix, Moutan Radicis Cortex, Carthami Flos, Glycyrrhizae Radix et Rhizoma |
| Nishioka 2011 | CIPN | Rehmanniae Radix Preparata, Corni Fructus, Dioscoreae Rhizoma, Moutan Radicis Cortex, Poria Sclerotium, Alismatis Rhizoma, Achyranthis Radix, Plantaginis |

| | | |
|---|---|---|
| | | Semen |
| Huang 2013 | CIPN | Angelicae Gigantis Radix, Cinnamomi Ramulus, Paeoniae Radix, Astragali Radix, Tetrapanacis Medulla, Zizyphi Fructus, Asiasari Radix et Rhizoma, Cnidii Rhizoma, Spatholobi Caulis, Scorpio, Lumbricus, Glycyrrhizae Radix et Rhizoma |
| Abe 2013 | CIPN | Rehmanniae Radix Preparata, Corni Fructus, Dioscoreae Rhizoma, Moutan Radicis Cortex, Poria Sclerotium, Alismatis Rhizoma, Achyranthis Radix, Plantaginis Semen |
| Kono 2013 | CIPN | Rehmanniae Radix Preparata, Corni Fructus, Dioscoreae Rhizoma, Moutan Radicis Cortex, Poria Sclerotium, Alismatis Rhizoma, Achyranthis Radix, Plantaginis Semen |
| Li 2013 | CIPN | Astragali Radix, Cinnamomi Ramulus, Paeoniae Radix, Spatholobi Caulis, Atractylodis Rhizoma Alba, Cnidii Rhizoma, Angelicae Gigantis Radix, Asiasari Radix et Rhizoma, Corydalis Tuber, Scorpio, Citri Unshius Pericarpium Immaturus, Chaenomelis Fructus, Achyranthis Radix, Glycyrrhizae Radix et Rhizoma |
| Oki 2015 | CIPN | Rehmanniae Radix Preparata, Corni Fructus, Dioscoreae Rhizoma, Moutan Radicis Cortex, Poria Sclerotium, Alismatis Rhizoma, Achyranthis Radix, Plantaginis Semen |

| | | |
|---|---|---|
| Xu 2017 | CIPN | Astragali Radix, Spatholobi Caulis, Codonopsis Pilosulae Radix, Cinnamomi Ramulus, Angelicae Gigantis Radix, Asiasari Radix et Rhizoma, Glycyrrhizae Radix et Rhizoma, Zingiberis Rhizoma Recens, Zizyphi Fructus |
| Xie 2018 | CIPN | Astragali Radix, Paeoniae Radix, Carthami Flos, Angelicae Gigantis Radix, Atractylodis Rhizoma Alba, Myrrha, Rehmanniae Radix Preparata, Dioscoreae Rhizoma, Lumbricus, Cnidii Rhizoma, Glycyrrhizae Radix et Rhizoma |
| Liu 2018 | CIPN | Astragali Radix, Paeoniae Radix, Zizyphi Fructus, Cinnamomi Ramulus, Zingiberis Rhizoma Recens |
| Zhang 2018 | CIPN | Astragali Radix, Lycii Fructus, Rehmanniae Radix Recens, Spatholobi Caulis, Cinnamomi Ramulus, Lumbricus, Carthami Flos, Asiasari Radix et Rhizoma, Puerariae Radix, Achyranthis Radix |
| Li 2020 | CIPN | Astragali Radix, Paeoniae Radix, Cinnamomi Ramulus, Zizyphi Fructus, Zingiberis Rhizoma Recens, Achyranthis Radix, Chaenomelis Fructus, Eucommiae Cortex, Mori Ramulus, Curcumae Longae Rhizoma, Rehmanniae Radix Preparata, Angelicae Gigantis Radix, Pinelliae Tuber |
| Liu 2020 | CIPN | Rehmanniae Radix Preparata, Corni Fructus, Psoraleae Semen, Cuscutae Semen, Angelicae Gigantis Radix, Cnidii Rhizoma, Rubi Fructus, Paeoniae Radix, Myrrha, Salviae Miltiorrhizae Radix, Lumbricus, Batryticatus Bombyx, Citri Unshius |

| | | |
|---|---|---|
| | | Pericarpium |
| Li 2016c | PHN | Forsythiae Fructus, Taraxaci Herba, Paris polyphylla, Gentianae Scabrae Radix et Rhizoma, Trichosanthis Radix, Angelicae Gigantis Radix, Thujae Semen, Zanthoxyli Pericarpium, Persicae Semen, Olibanum, Myrrha, Corydalis Tuber, Pseudostellariae Radix, Poria Sclertum Cum Pini Radix, Glycyrrhizae Radix et Rhizoma, Plantaginis Semen, Atractylodis Rhizoma, Agastachis Herba, Amomi Fructus Rotundus, Eupatorii Herba, Artemisiae Capillaris Herba |
| Zhang 2012 | PHN | Saposhnikoviae Radix, Magnoliae Cortex, Citri Unshius Pericarpium, Poria Sclerotium, Atractylodis Rhizoma, Atractylodis Rhizoma Alba, Angelicae Gigantis Radix, Salviae Miltiorrhizae Radix, Spatholobi Caulis, Liquidambaris Fructus, Lumbricus, Corydalis Tuber, Meliae Fructus, Linderae Radix |
| Zhao 2018 | PHN | Bupleuri Radix, Corydalis Tuber, Astragali Radix, Codonopsis Pilosulae Radix, Rehmanniae Radix Preparata, Angelicae Gigantis Radix, Salviae Miltiorrhizae Radix, Cnidii Rhizoma, Carthami Flos, Olibanum, Myrrha, Glycyrrhizae Radix et Rhizoma |
| Gong 2021 | Occiptal neuralgia | Cnidii Rhizoma, Schizonepetae Spica, Angelicae Dahuricae Radix, Osterici seu Notopterygii Radix et Rhizoma, Asiasari Radix et Rhizoma, Saposhnikoviae Radix, Menthae Herba, Gastrodiae Rhizoma, Achyranthis Radix, Nardotidis seu Sulculii |

| | | |
|---|---|---|
| | | Concha, Uncariae Ramulus cum Uncus, Citri Unshius Pericarpium, Pinelliae Tuber, Atractylodis Rhizoma Alba, Scorpio, Batryticatus Bombyx, Salviae Miltiorrhizae Radix |
| Huang 2020 | Trigeminal neuralgia | Cnidii Rhizoma, Ilex pubescens, Angelicae Dahuricae Radix, Saposhnikoviae Radix, Osterici seu Notopterygii Radix et Rhizoma, Angelicae Gigantis Radix, Scorpio, Glycyrrhizae Radix et Rhizoma, Astragali Radix, Atractylodis Rhizoma Alba, Codonopsis Pilosulae Radix, Liriopis seu Ophiopogonis Tuber, Rehmanniae Radix Recens, Prunellae Spica, Gentianae Scabrae Radix et Rhizoma, Poria Sclerotium, Alismatis Rhizoma, Persicae Semen, Carthami Flos, Aconiti Lateralis Radix Preparata, Albizziae Cortex, Polygoni Multiflori Radix |
| Song 2020 | Supraorbital neuralgia | Rehmanniae Radix Preparata, Paeoniae Radix, Angelicae Gigantis Radix, Cnidii Rhizoma, Angelicae Dahuricae Radix, Saposhnikoviae Radix, Ligustici Tenuissimi Rhizoma et Radix, Buddlejae Flos, Prunellae Spica, Cyperi Rhizoma |
